# Supplementary figures and images for: Sulfonylpiperazine compounds prevent Plasmodium falciparum invasion of red blood cells through interference with actin-1/profilin dynamics
Source: PLoS Biol. 2023 Apr 13;21(4):e3002066. doi: 10.1371/journal.pbio.3002066 (PMC10128974; doi:10.1371/journal.pbio.3002066)

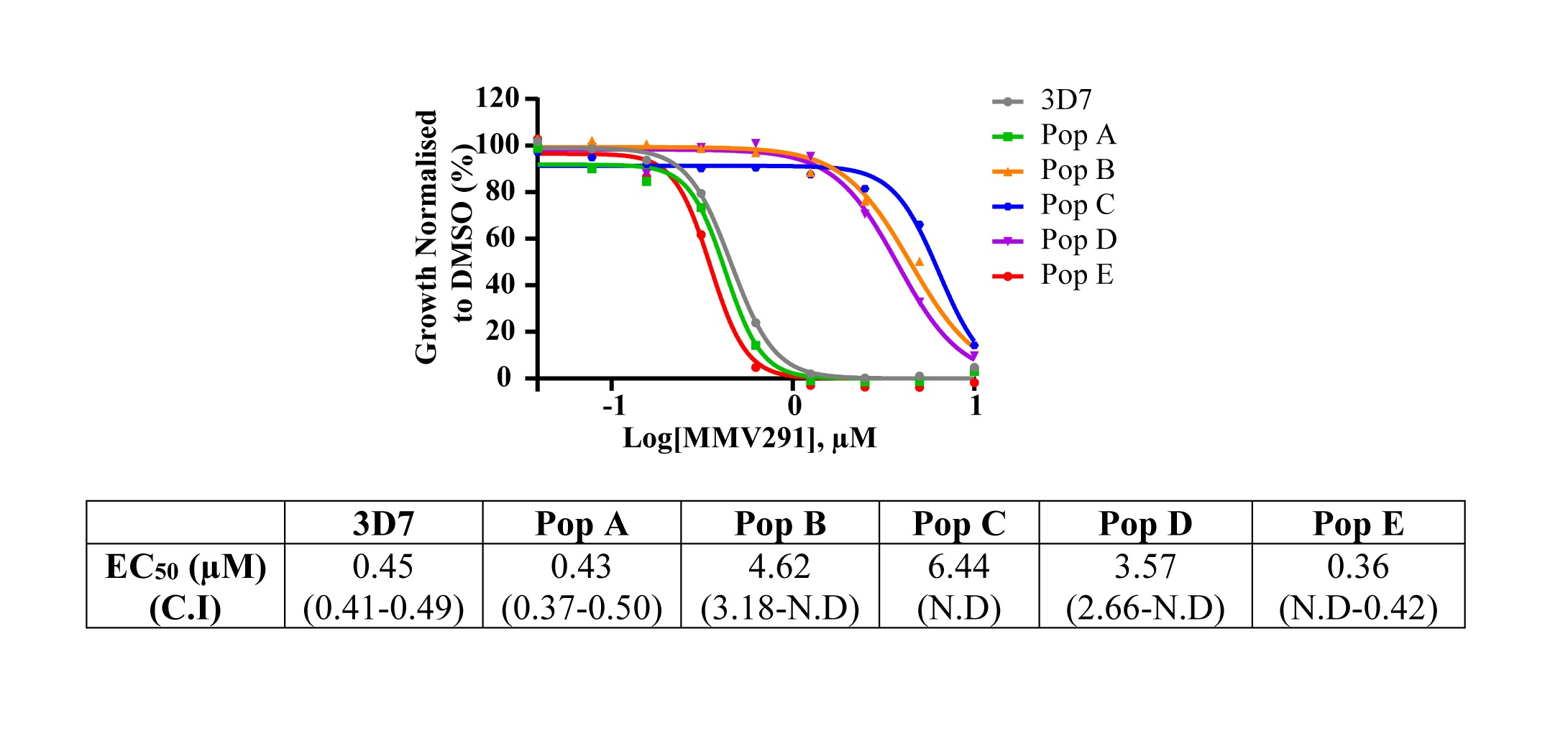

Supplement: S1 Fig — Viable parasites recovered after 3 rounds of drug cycling were tested against a titration of MMV291 in a 72-hour lactate dehydrogenase (LDH) growth assay. Parasite growth was normalised to parasite’s grown in 0.1% DMSO, which indicated 3 resistant populations were obtained (B, C, and D) with an 8- to 14-fold increase in EC50 compared to 3D7. Data points represent the average of 3 technical replicates. C.I indicates 95% confidence intervals for EC50 values, which were derived from nonlinear regression curves in GraphPad Prism. N.D = not determined. Source data can be found in S1 Data. (TIF) [file pbio.3002066.s001.tif]

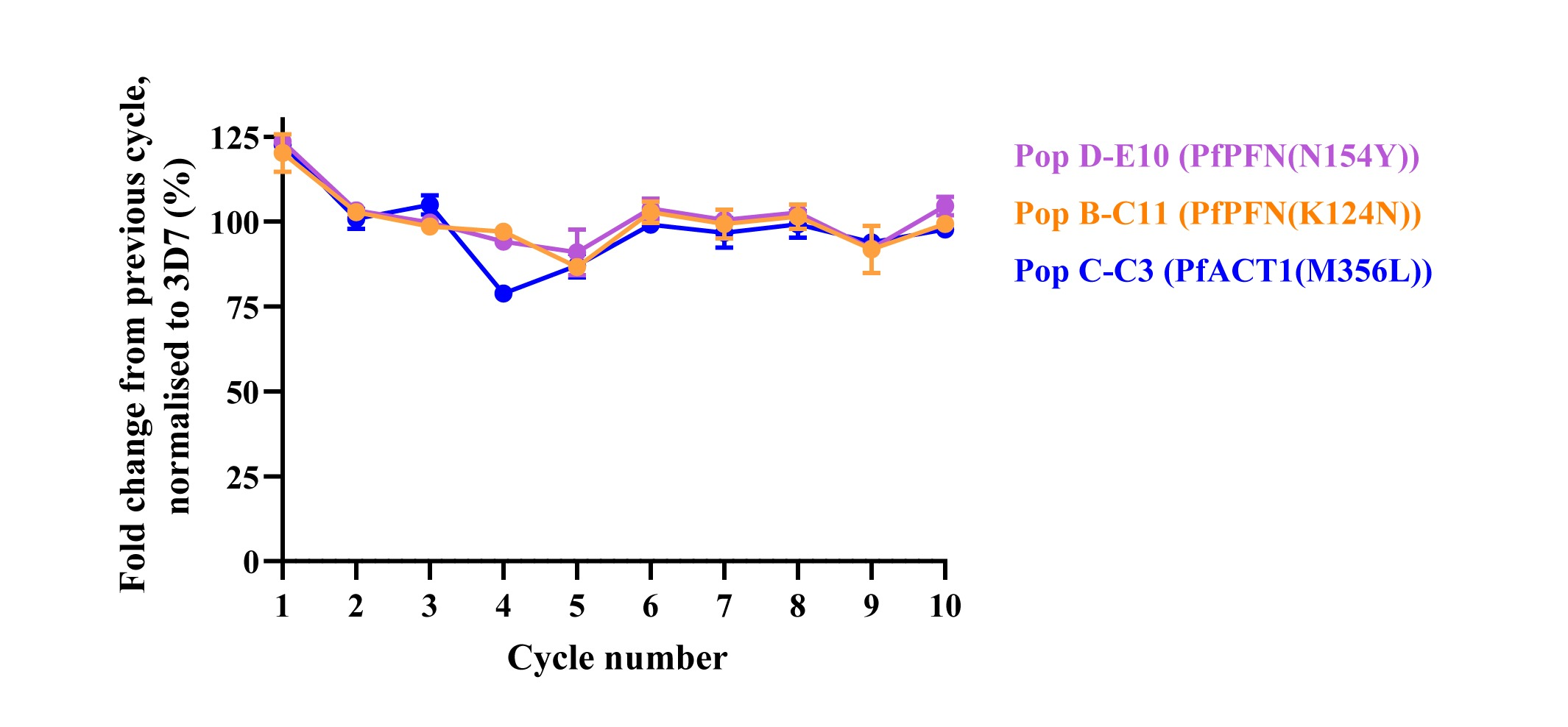

Supplement: S2 Fig — The growth of 3 MMV291-resistant population clones, Pop D-E10, Pop B-C11, and Pop C-C3, with the corresponding PFN(N154Y), PFN(K124N), and ACT1(M356L) mutations, along with 3D7 WT parasites, were compared in a 10-cycle growth assay. During each cycle, an aliquot of culture was harvested from each parasite line and frozen until completion of the assay, whereby parasite lactate dehydrogenase was measured as a marker for parasite growth. The fold change in parasitemia was calculated from the previous cycle for each parasite line, which was then expressed as a percentage of the 3D7 fold change. This demonstrated that there was no comparative growth defect associated with the resistant lines, indicating that the mutations in profilin and actin-1 did not reduce the fitness of these parasites. Error bars represents the standard deviation from 1 experiment comprising of 3 technical replicates. Source data can be found in S1 Data. (TIF) [file pbio.3002066.s002.tif]

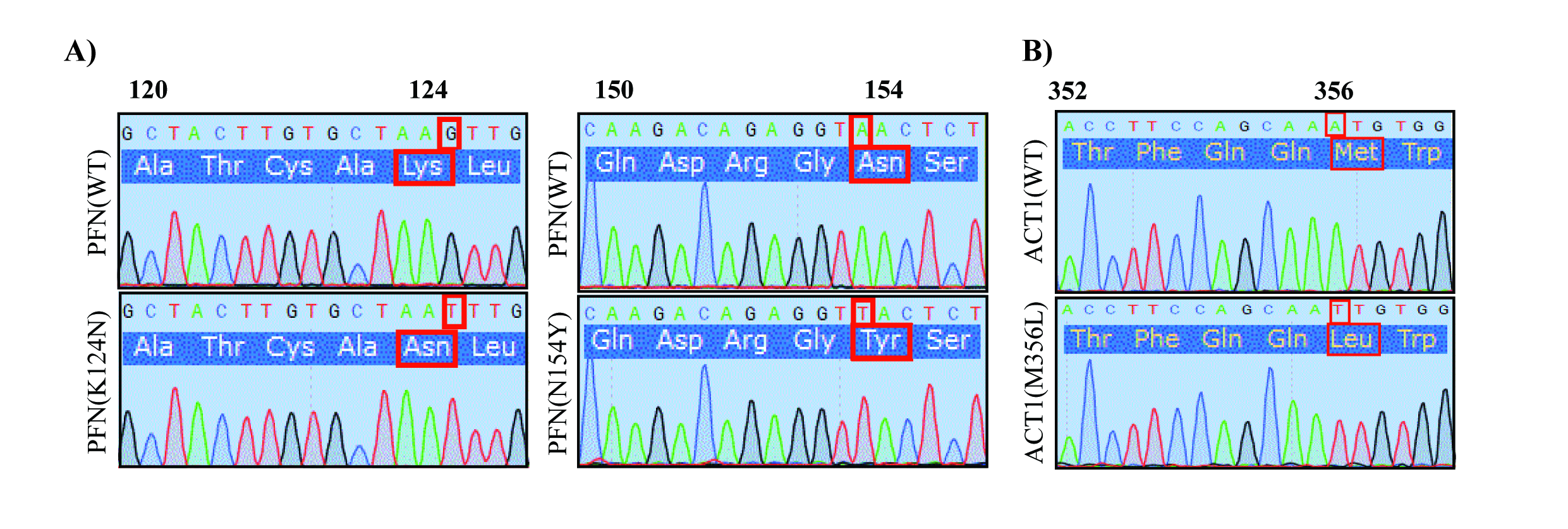

Supplement: S3 Fig — The products produced from diagnostic PCRs were sequenced, and the resistant mutations were confirmed to be present for (A) K124N (AAG-AAT) and N154Y (AAC-TAC) in profilin and (B) M356L (ATG-TTG) in actin-1. (TIF) [file pbio.3002066.s003.tif]

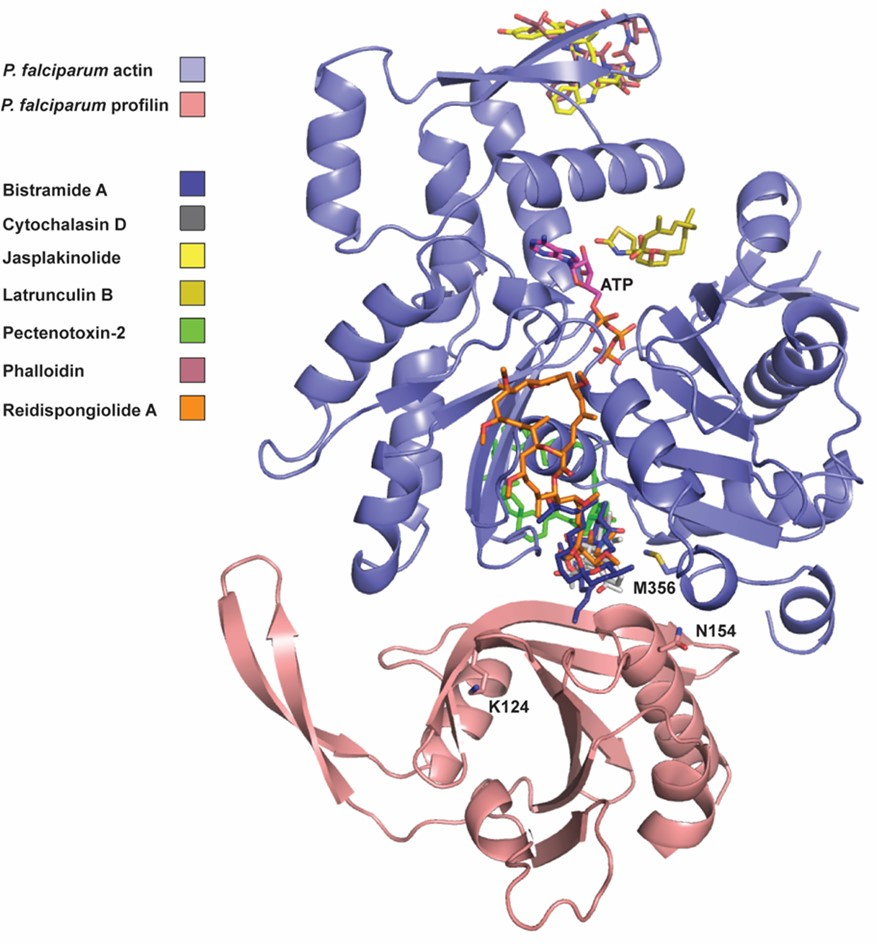

Supplement: S4 Fig — P. falciparum profilin (pink) (PDB: 2JKG) ([36]; structure 16: 1638) and actin-1 (blue) (ATP, magenta) (PDB: 6I4E) [42] heterodimeric complex showing regions of the proteins where actin inhibitors are known to bind relative to the MMV291 P. falciparum mutations. The actin inhibitors aligned to P. falciparum actin-1 and shown are Bistramide A (blue) (aligned from O. cuniculus actin, PDB: 2FXU) [101], Cytochalasin D (grey) (aligned from D. melanogaster actin, PDB: 3EKU) [102], Jasplakinolide (yellow) (aligned from P. falciparum F-actin, PDB: 5OGW) [48], Latrunculin B (gold) and Pectenotoxin-2 (green) (aligned from O. cuniculus actin, PDB: 2Q0U) [103], Phalloidin (maroon) (aligned from G. gallus F-actin, PDB: 7BTI) [104], and Reidispongiolide A (orange) (aligned from O. cuniculus actin PDB: 2ASM) [105]. (TIF) [file pbio.3002066.s004.tif]

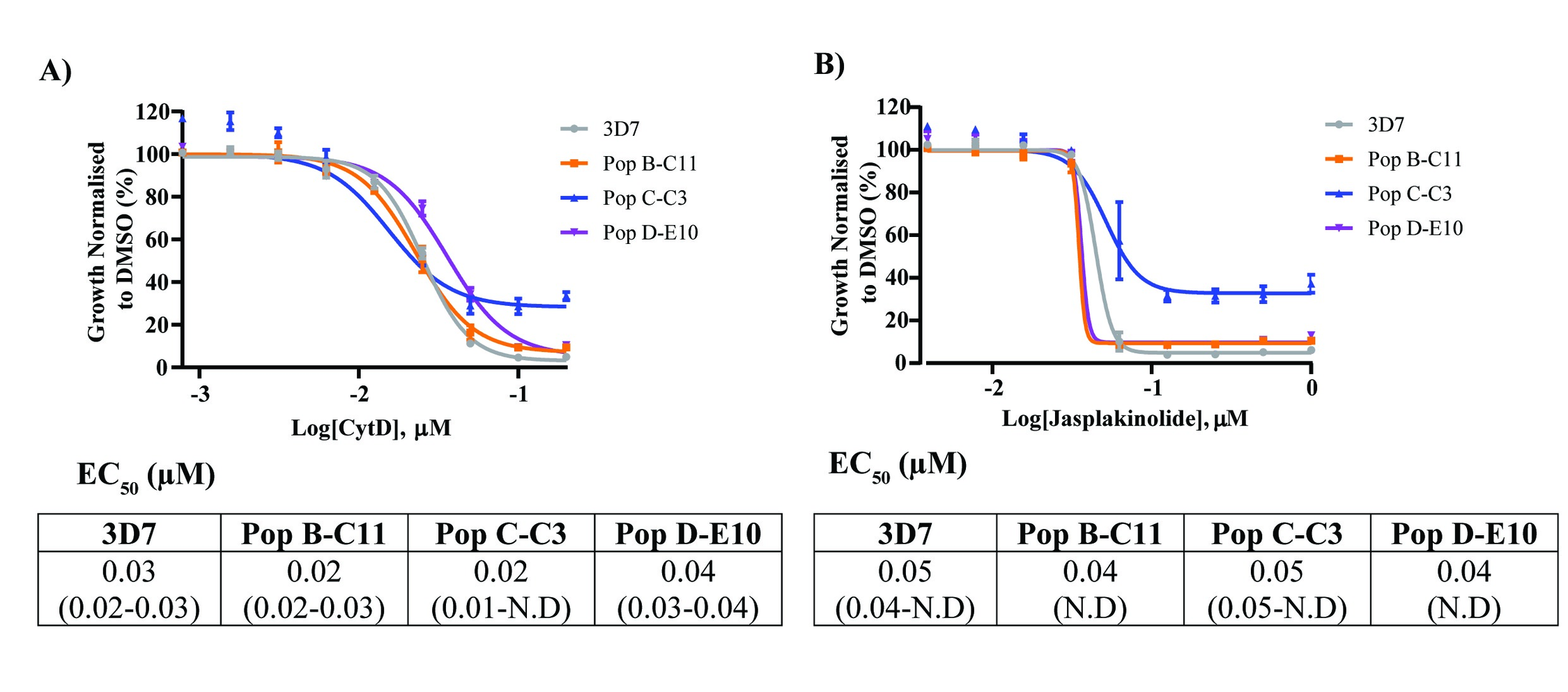

Supplement: S5 Fig — A titration of the actin polymerisation inhibitor, Cytochalasin D (CytD) (A), and actin polymerisation stabiliser, Jasplakinolide (B), were tested against the MMV291-resistant lines and 3D7 parasites in a 72-hour growth assay. This revealed that MMV291-resistant parasites did not exhibit cross resistance to CytD and Jasplakinolide, indicating that MMV291 has an alternate mechanism of action. Growth has been normalised to that of parasites grown in 0.1% DMSO, and error bars represent the standard deviation of 3 biological replicates. EC50 values were derived from nonlinear regression curves in GraphPad Prism with 95% confidence intervals of these values specified in brackets. Source data can be found in S1 Data. (TIF) [file pbio.3002066.s005.tif]

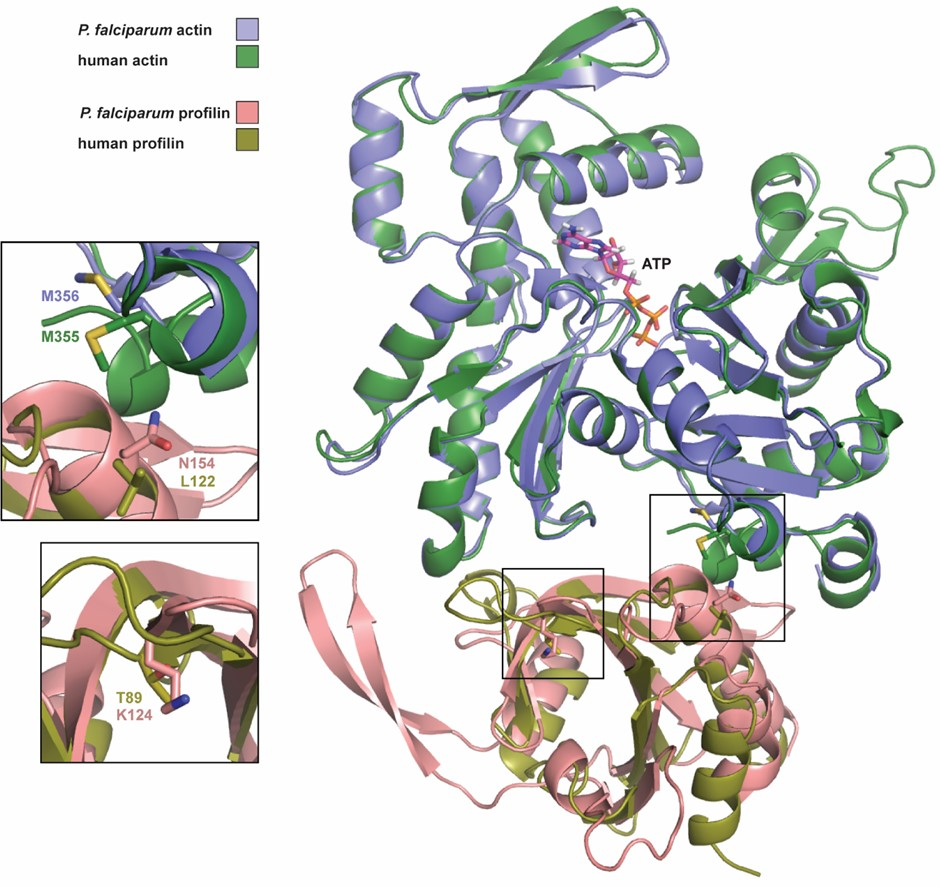

Supplement: S6 Fig — An X-ray structure human profilin (gold) (PDB: 2PBD) and a homology model of human actin (green) (created by SWISS-MODEL [106] using O. cuniculus actin (PDB: 2PBD) [56] aligned with P. falciparum profilin (pink) (PDB: 2JKG) [36] and actin (blue) (PDB: 6I4E) [42] showing the similarity of the heterodimeric complex. The positions of the MMV291 P. falciparum mutations and the associated human amino acids are shown for comparison. (TIF) [file pbio.3002066.s006.tif]

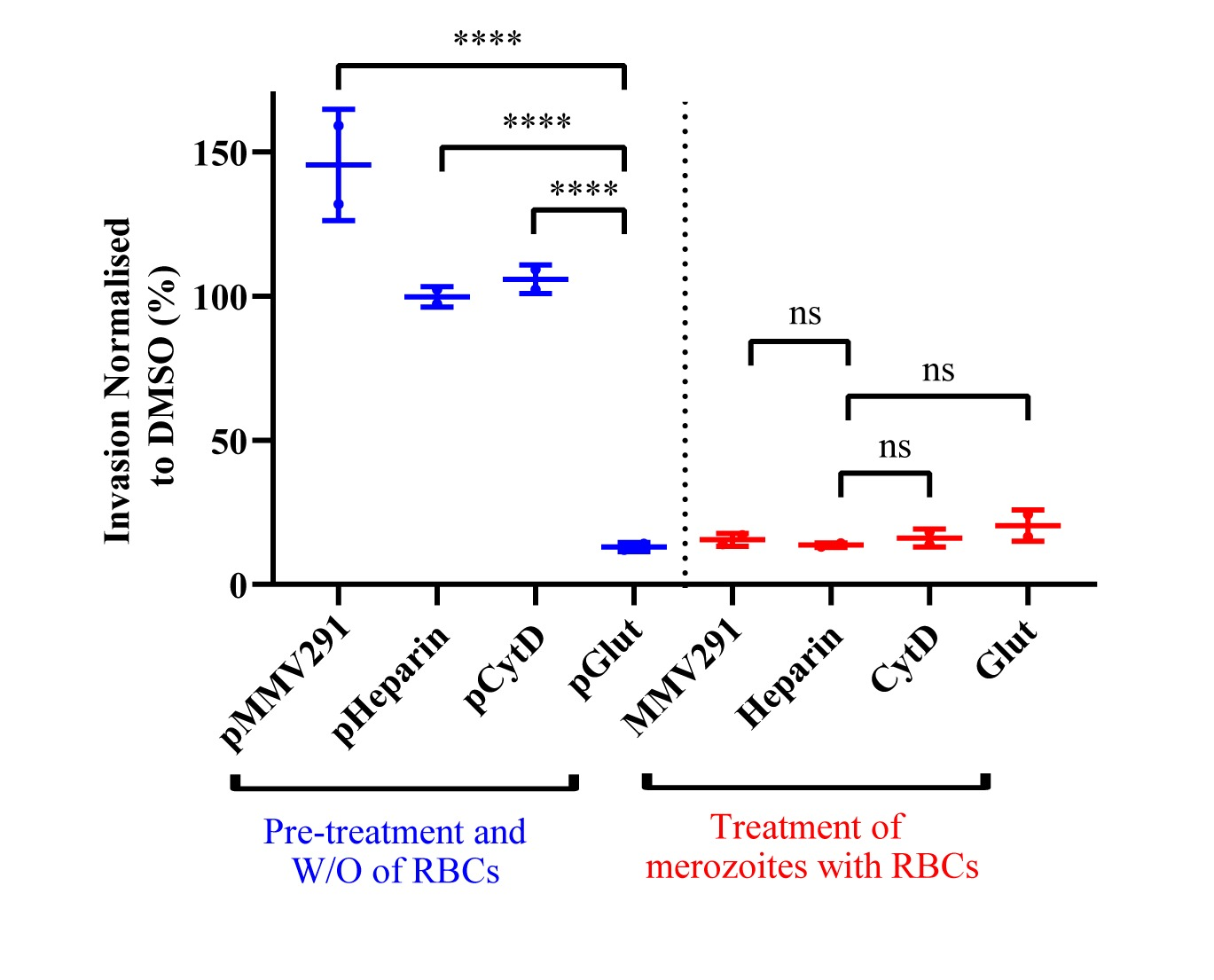

Supplement: S7 Fig — Uninfected RBCs were pretreated with invasion inhibitory compounds (blue); 10 μM MMV291, 100 μg/mL heparin, 2 μM cytochalasin D (CytD), or 0.0025% glutaraldehyde (Glut) for 30 minutes at 37°C, after which the cells were washed (W/O) to remove the inhibitors. Purified merozoites were then allowed to invade the pretreated RBCs. In parallel, merozoites were added to untreated RBCs in the presence of these inhibitors (red). After incubation for 30 minutes at 37°C, the compounds were washed out and parasites allowed to grow for 24 hours. Successful invasion was assessed by measuring the bioluminescence levels of trophozoite-stage parasites expressing a nanoluciferase reporter, and invasion rate was normalised to the DMSO vehicle control. This demonstrated that unlike the fixative glutaraldehyde, pretreatment with MMV291 did not reduce merozoite invasion of RBCs, producing a similar profile to the invasion inhibitory molecules, heparin and CytD. Error bars represent the standard deviation of 2 biological replicates with statistical analyses performed in GraphPad Prism using a one-way ANOVA with pretreated RBCs compared to glutaraldehyde (blue) and merozoite treatment compared to heparin (red). **** indicates P < 0.0001; ns indicates not significant (P > 0.05). Source data can be found in S1 Data. (TIF) [file pbio.3002066.s007.tif]

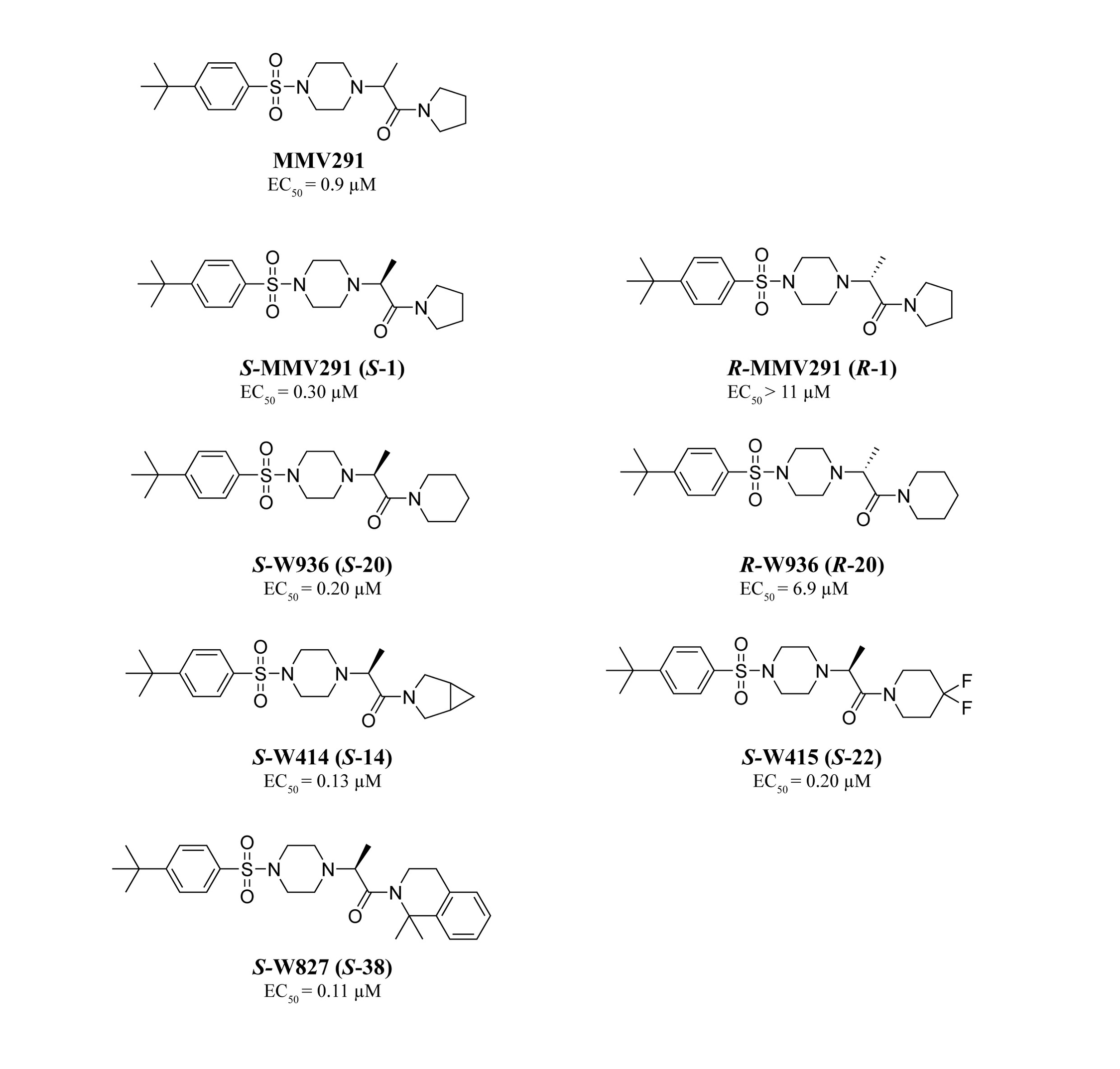

Supplement: S8 Fig — The chemical structures and corresponding EC50 values against the RBC stage of P. falciparum used in this study with original compound names from Nguyen and colleagues (2021) [53] specified in brackets. (TIF) [file pbio.3002066.s008.tif]

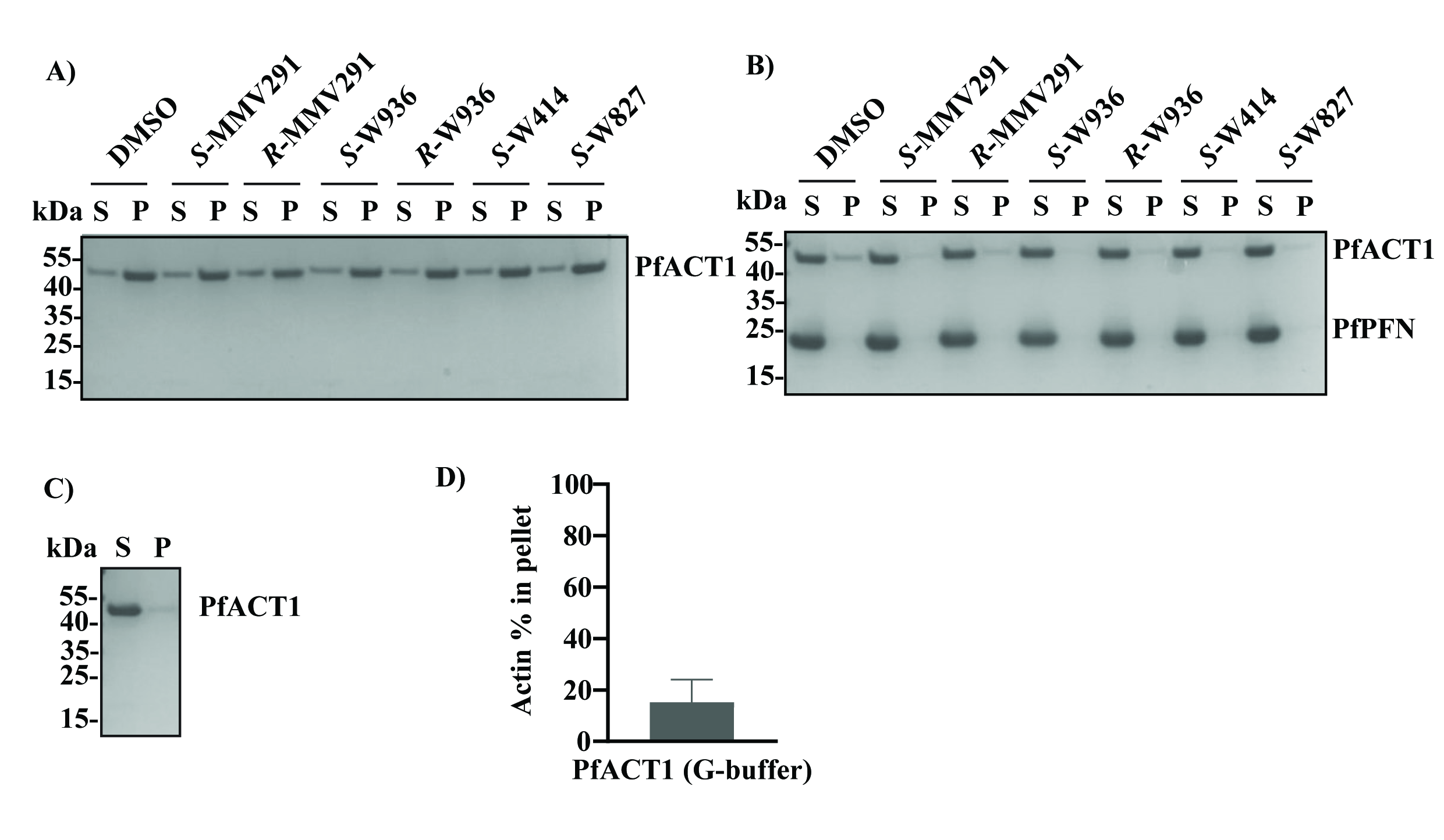

Supplement: S9 Fig — Sedimentation samples consisting of 4 μM PfACT1 (A) and with 16 μM PfPFN (B) in the presence of 25 μM MMV291 analogues or DMSO as well as 4 μM PfACT1 alone in G-buffer (C) were analysed on 4%–20% Mini-PROTEAN TGX gels and visualized with PageBlue stain. S denotes supernatant and P pellet. D) Quantification of the relative amount of actin in the pellet fraction for PfACT1 in G-buffer. 15 ± 9% of PfACT1 sedimented to the pellet fraction in G-buffer. Results are reported as mean ± standard deviation. Source data can be found in S1 Data. The data are based on at least 3 independent assays each performed in triplicate, with a representative gel presented. (TIF) [file pbio.3002066.s009.tif]

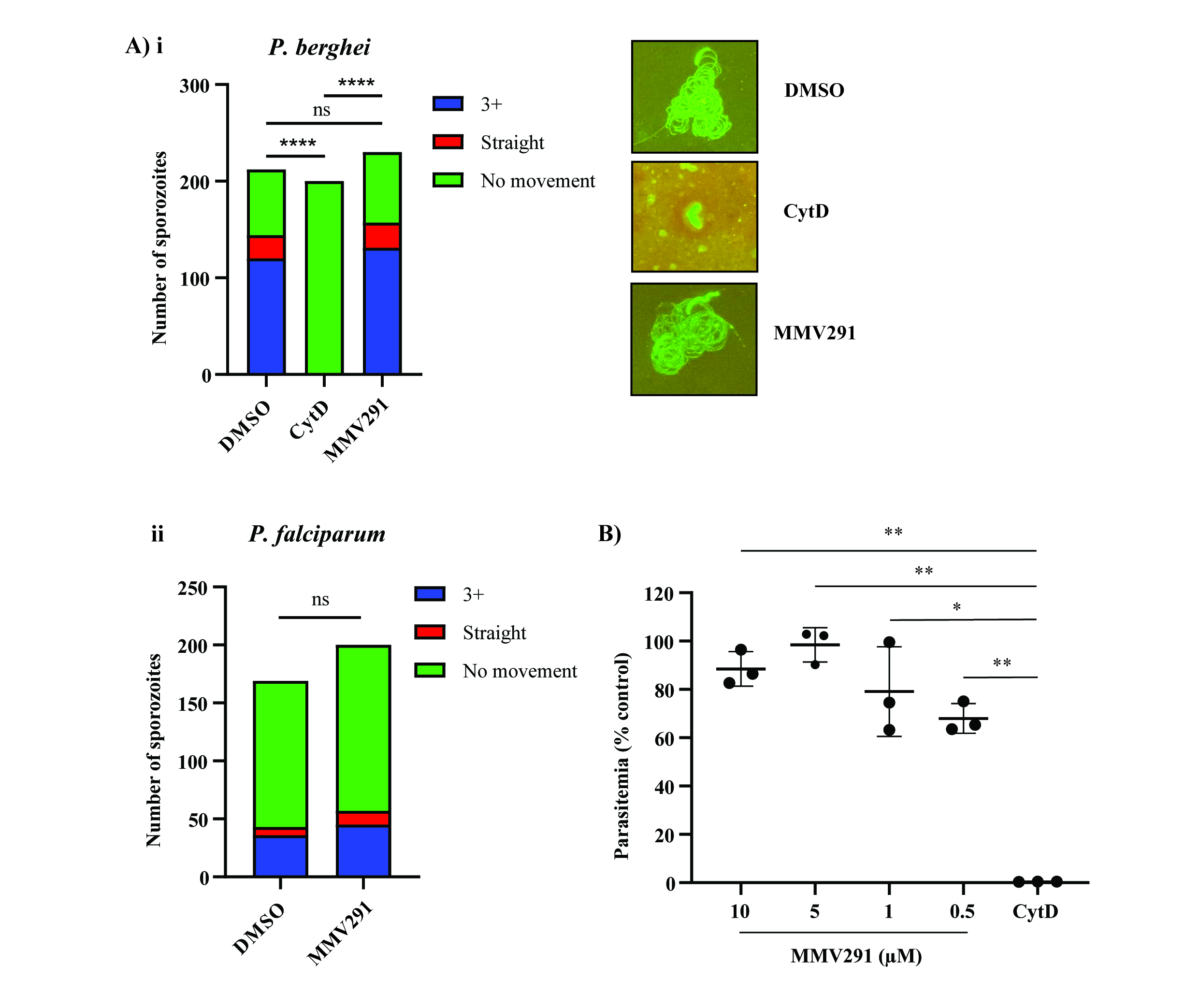

Supplement: S10 Fig — (A) Sporozoites expressing GFP were used to measure motility via the quantification of fluorescent trails. This revealed that similarly to DMSO, MMV291 had no effect on sporozoite motility in P. berghei (i) or P. falciparum (ii), while cytochalasin D (CytD) significantly reduced motility. 3+ indicates 3 or more trails observed. (B) In vitro human liver cells were incubated with a titration of MMV291 in the presence of 20,000 sporozoites expressing a luciferase protein. After 52 hours, cells were lysed and luciferase activity was measured to correlate with sporozoite invasion rate. In contrast with CytD (10 μM) treatment, MMV291 did not reduce invasion rate of sporozoites at concentrations tested. Error bars represent the standard deviation across 3 biological replicates each comprised of 3 technical replicates. Statistical analysis performed via a chi-squared (A) and unpaired t test (B) using GraphPad Prism. * P < 0.05, ** P < 0.01, **** P < 0.0001; ns indicates not significant. Source data can be found in S1 Data. (TIF) [file pbio.3002066.s010.tif]

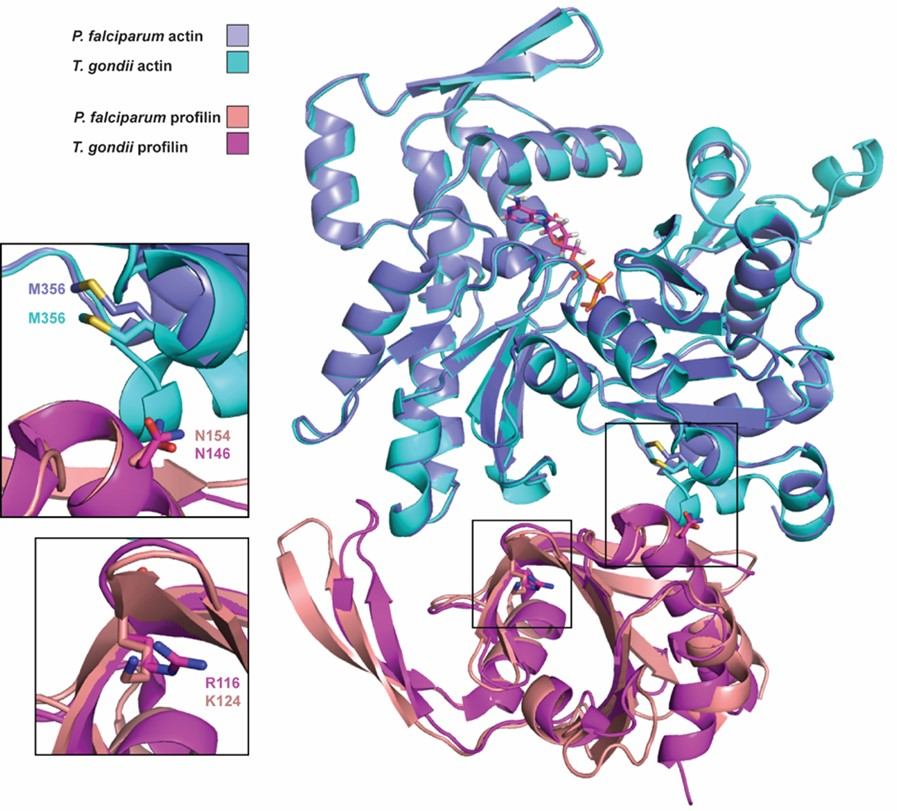

Supplement: S11 Fig — T. gondii profilin (magenta) and actin (cyan) aligned with P. falciparum profilin (pink) and actin (blue) showing the similarity of the heterodimeric complex and the positions of the MMV291 P. falciparum mutations. The X-ray structure of T. gondii profilin (PDB: 3NEC) [85] and a homology model of T. gondii actin (created by SWISS-MODEL [106] using the X-ray structure of P. falciparum actin (PDB: 6I4K) [42] were used to create the model. The X-ray structure of O. cuniculus actin and human profilin (PDB: 2PBD) [56] was utilised as a template to spatially overlay the P. falciparum actin and profilin in the heterodimer model. (TIF) [file pbio.3002066.s011.tif]

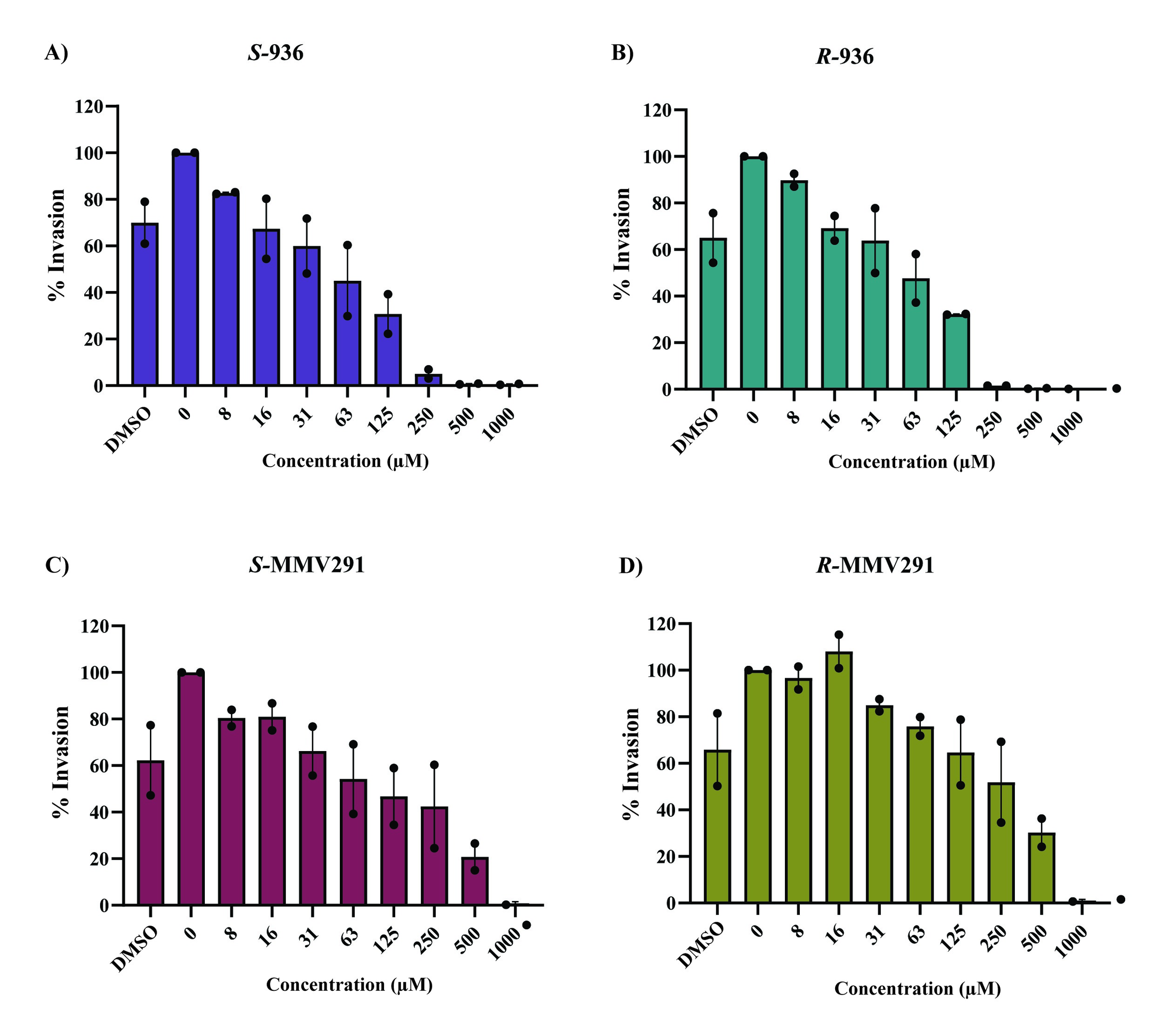

Supplement: S12 Fig — Nanoluciferase expressing parasites were liberated from their host cell and incubated with the MMV291 analogues before being added back to fibroblasts and allowed to invade for 1 hour before compounds were washed out. After a 24-hour incubation, cells were then lysed and the relative light units was quantified to correlate with T. gondii invasion rate. This showed MMV291 analogues S-W936 (A), R-W936 (B), S-MMV291 (C), and R-MMV291 (D) had some inhibitory activity against invasion at high concentrations. DMSO was included to the same amount as the highest concentration of analogue to account for DMSO-related effects (30% reduction in invasion). Error bars indicate the standard deviation from 2 biological repeats. Source data can be found in S1 Data. (TIF) [file pbio.3002066.s012.tif]

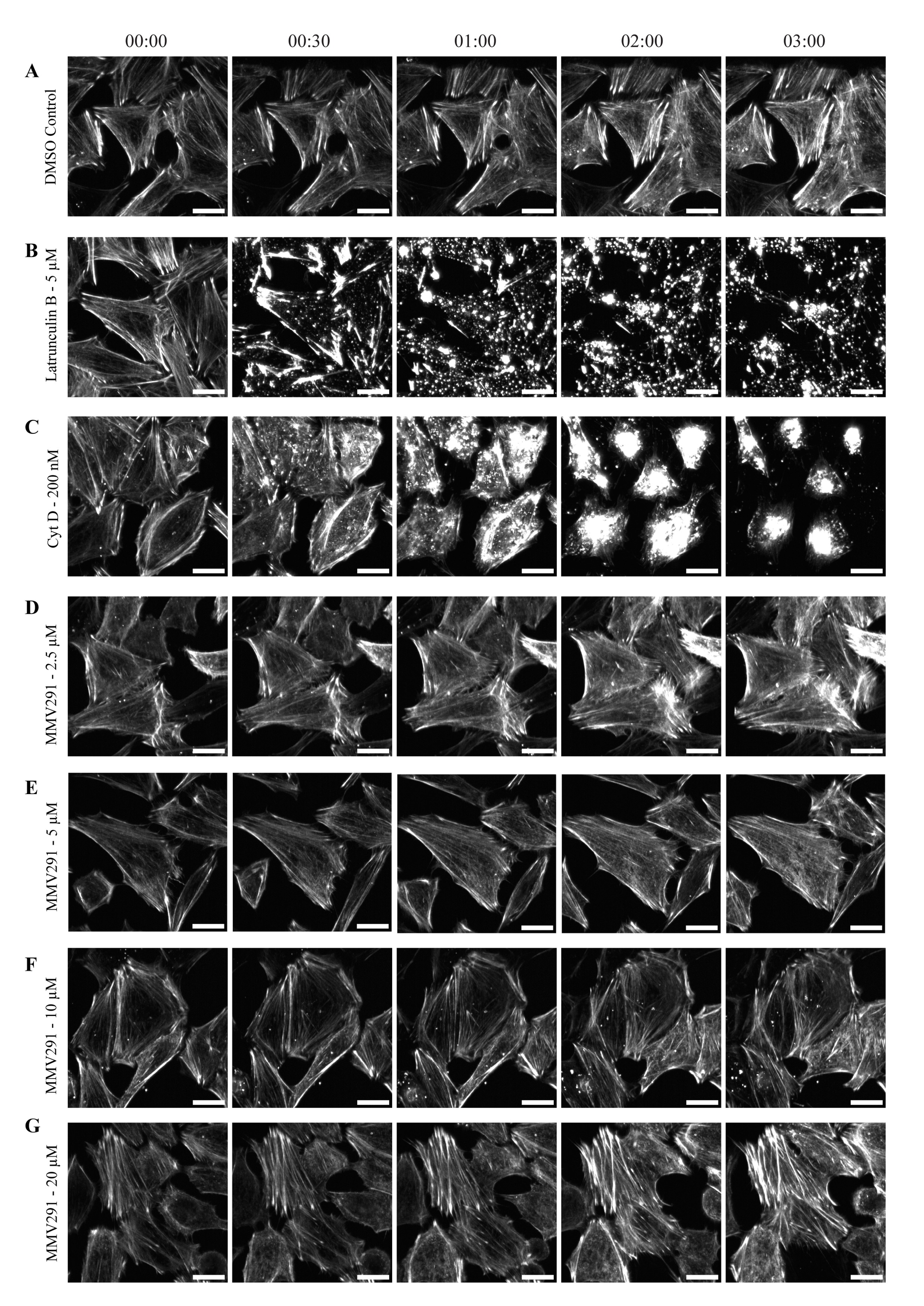

Supplement: S13 Fig — HeLa cells labelled with SiR-Actin imaged by lattice light-sheet microscopy upon stimulation with DMSO control (A), 5 μM Latrunculin B (B), 200 nM Cytochalasin D (CytD) (C), 2.5 μM MMV291 (D), 5 μM MMV291 (E), 10 μM MMV291 (F), and 20 μM MMV291 (G). Images represent a 100 × 100 μm subregion of a larger 250 × 250 μm field of view. Images are presented as maximum intensity projections with the contrast scaled between 100–400 counts. The images show the same region of cells imaged across multiple time points. Time is presented as HH:MM, and the scale bar represents 20 μm. (TIF) [file pbio.3002066.s013.tif]
