# Supplementary material for: Sulfonylpiperazine compounds prevent Plasmodium falciparum invasion of red blood cells through interference with actin-1/profilin dynamics
Source: PLoS Biol. 2023 Apr 13;21(4):e3002066. doi: 10.1371/journal.pbio.3002066 (PMC10128974; doi:10.1371/journal.pbio.3002066)
Supplement: S1 Table — (DOCX) [file pbio.3002066.s015.docx]

| **To knock-in mutations in Profilin** | | | |
| --- | --- | --- | --- |
|  |  |  |  |
| HR1A | PFN_BglII.1F (a) | *AGATCT*TTCATGGGACAGTTATTTAAATGATCGCCT | *Bgl*II |
|  | PFN_2R (b) | AGATAGTTTGACCTTCGTTGATCGTTTTGGTAGTTTTAGTACCA |  |
| HR1B | PFN_CO.3F (c) | CAACGAAGGTCAAACTATCTTGGTTGT |  |
|  | PFN_CO.Spe.4R (d) | *ACTAGT*CTATTGAGAAGATTCAGCCAATTCCTTAGCGA | *Spe*I |
| HR2 | PFN_EcoRI.5F (e) | *GAATTC*TGGTCGTTTTTAATGAAGGATATGCTCCTGA | *EcoR*I |
|  | PFN_KasI.6R (f) | *GGCGCC*CTATTGACTGCTTTCAGCTAGCTCTTTTGC | *Kas*I |
| gRNA | PFN_gRNA | AAATGAAGGACAAACGATCC/TGG |  |
| Introduce K124N | PFN_K12N_F (g) | TACTTGTGCTAATTTGAAGGGTGGT |  |
|  | PFN_K124N_R (h) | ACCACCCTTCAAATTAGCACAAGTA |  |
| Confirm integration | PFN_5'UTR_F (i) | CCTTTTCTTTTTCTTTCTTCCTCTCACTCTCTCATACTCTCT |  |
|  | PFN_WT_R (j) | TGGAGTTGTCTATGCTTGTGTAGCTCAGGG |  |
|  | glmS_R (k) | AGATCATGTGATTTCTCTTTGTTCA |  |
| **To knock-in mutations in Actin-1** | | | |
|  |  |  |  |
| HR1A | ACT_BglII.1F (l) | *AGATCT*AAAATGGGAGAAGAAGATGTTCAAGCT | *Bgl*II |
|  | ACT_2R (m) | GTCGAGACGCATAATCGCGTGTGGTAAAGCATAACCTTCATAAATTGGA |  |
| HR1B | ACT_CO.3F (n) | CACGCGATTATGCGTCTCGAC |  |
|  | ACT_CO.Spe.4R (o) | *ACTAGT*TTAGAAGCATTTACGATGGACAATAGA | *Spe*I |
| HR2 | ACT_EcoRI.5F (p) | *GAATTC*TTTAGCTGGTAGAGATTTAACTGAATATTTAATGA | *EcoR*I |
|  | ACT_KasI.6R (q) | *GGCGCC*TTAGAAACATTTTCTGTGGACAATACTTGGTCCT | *Kas*I |
| gRNA | ACT_sgRNA | TCTAATCTCATAATTGCATG/TGG |  |
| Introduce M356L | ACT_CO_M356L.F (r) | CTCTTTCCACCTTCCAGCAATTGTGGATTACT |  |
|  | ACT_CO_M356L.R (s) | AGTAATCCACAATTGCTGGAAGGTGGAAAGAG |  |
| Replace internal *Bgl*II site | ACT_3'_repairF (t) | GGATATTCGTAAAGACCTTTATGGAAATATCGT |  |
|  | ACT_5'_repairR (u) | ACGATATTTCCATAAAGGTCTTTACGAATATCC |  |
| Confirm integration | ACT_5'UTR_F (v) | CCATTTGGTGATTAGTTTTTACTGAC |  |
|  | ACT_WT_R (w) | GCTCCAGAAGAACACCCAGTGTTATTAAC |  |
|  | glmS_R (k) | AGATCATGTGATTTCTCTTTGTTCA |  |

**Table S1. Primers used to construct donor plasmids**

Italicised text indicates restriction enzyme sites and underlined text in guide RNA (gRNA) sequences indicate protospacer adjacent motif (PAM) sites.
