# Supplementary material for: Sulfonylpiperazine compounds prevent Plasmodium falciparum invasion of red blood cells through interference with actin-1/profilin dynamics
Source: PLoS Biol. 2023 Apr 13;21(4):e3002066. doi: 10.1371/journal.pbio.3002066 (PMC10128974; doi:10.1371/journal.pbio.3002066)

Figure 2 raw images

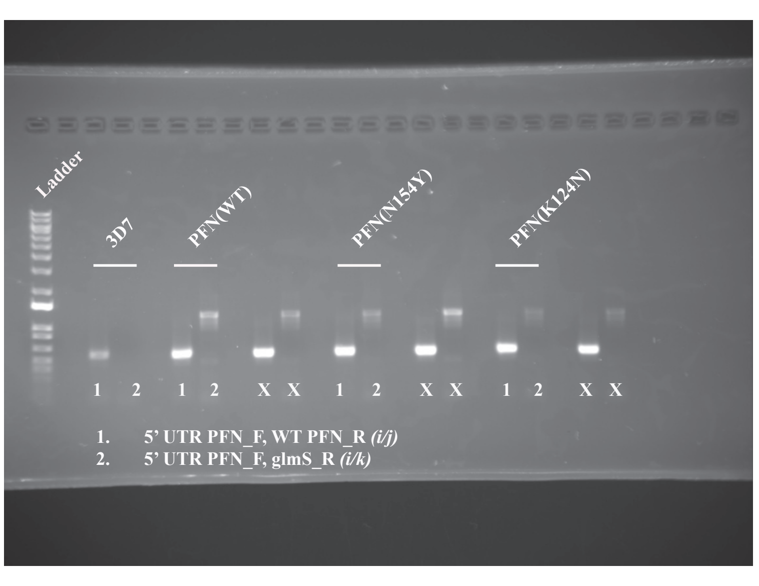

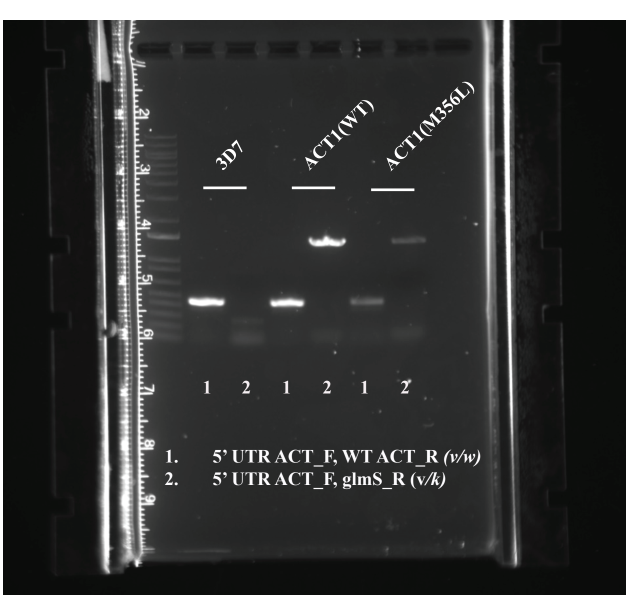

Figure 5 and S9 raw images

Experiment 1 gel 1

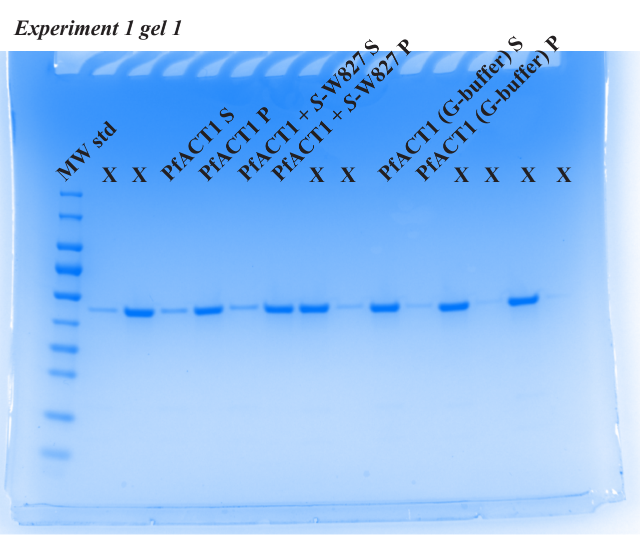

Experiment 1 gel 2

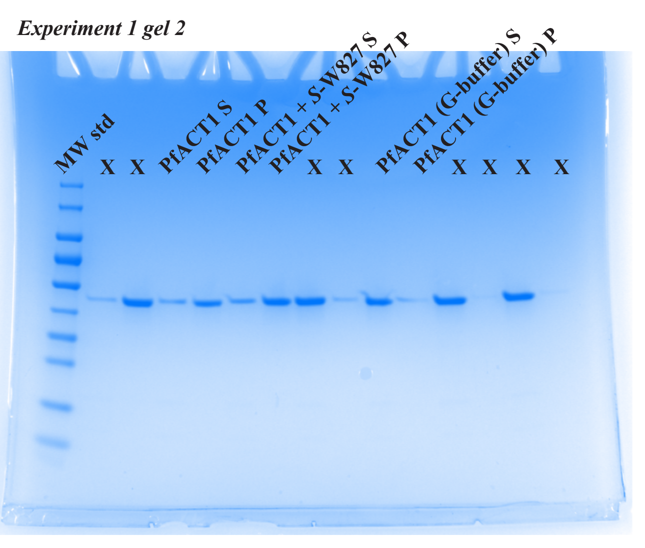

Experiment 1 gel 3

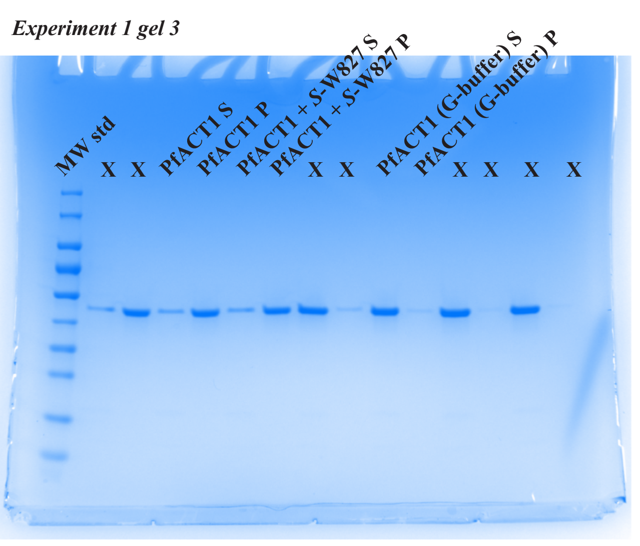

Experiment 2 gel 1

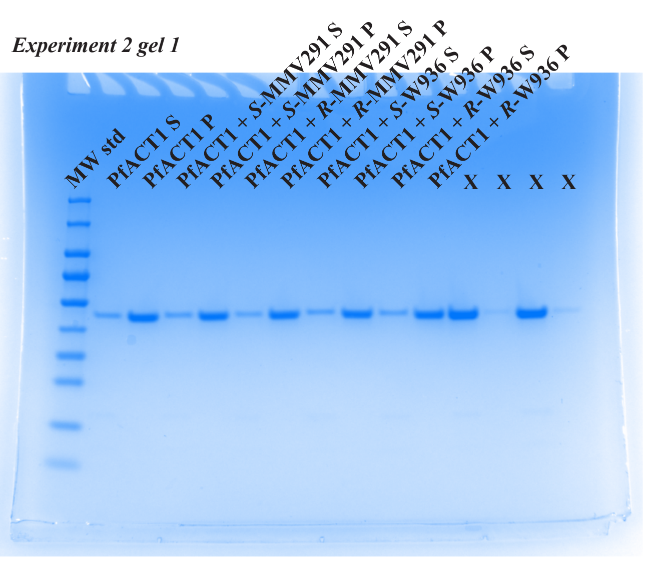

Experiment 2 gel 2

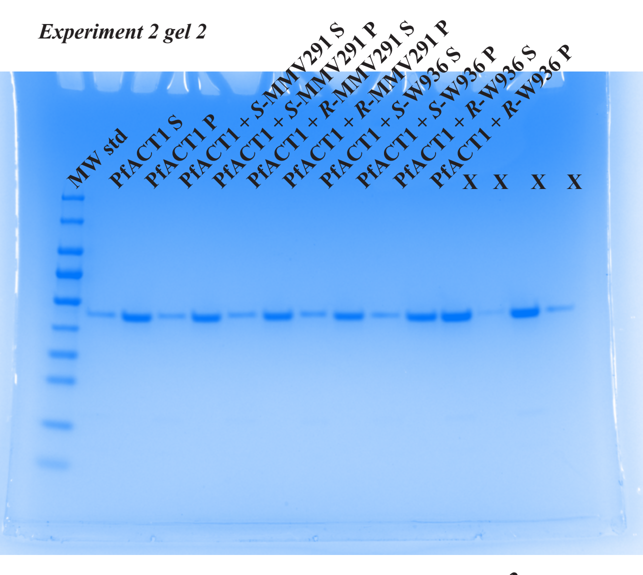

Experiment 2 gel 3

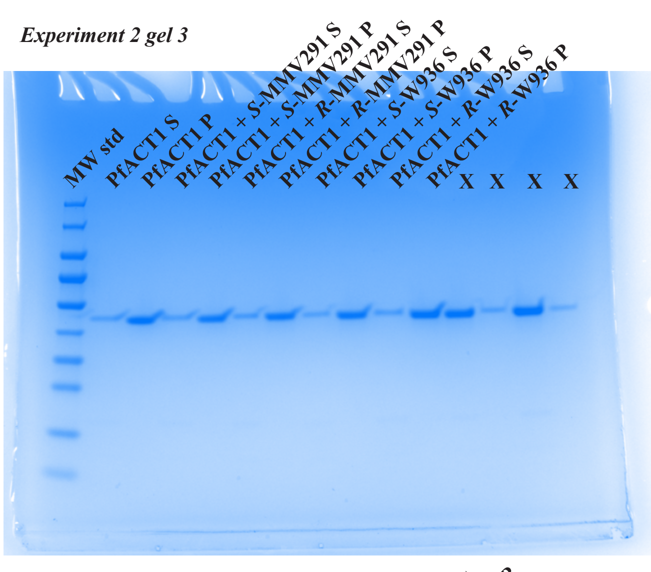

Experiment 2 gel 4

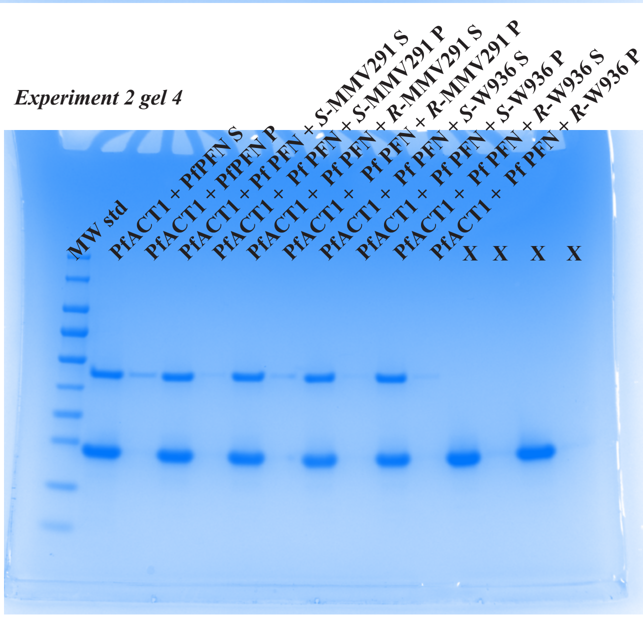

Experiment 2 gel 5

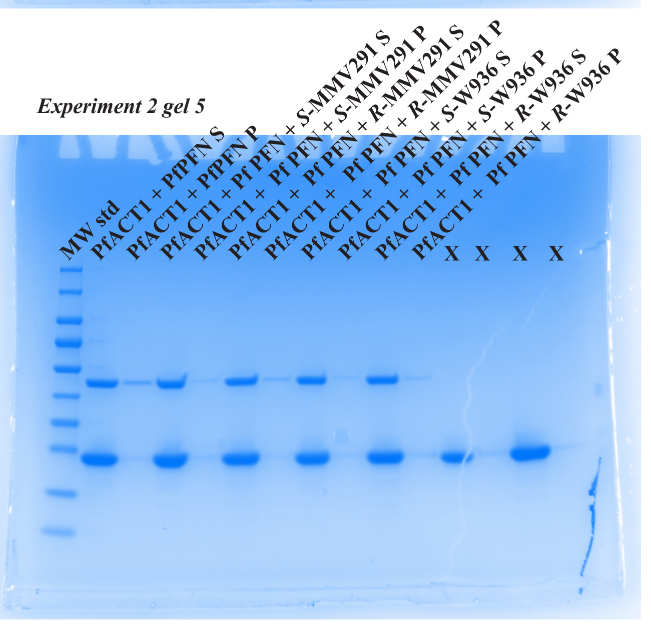

Experiment 2 gel 6

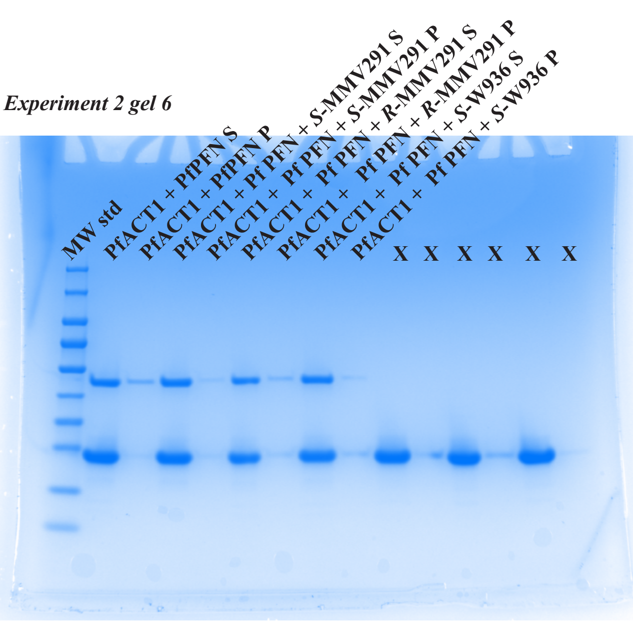

Experiment 2 gel 7

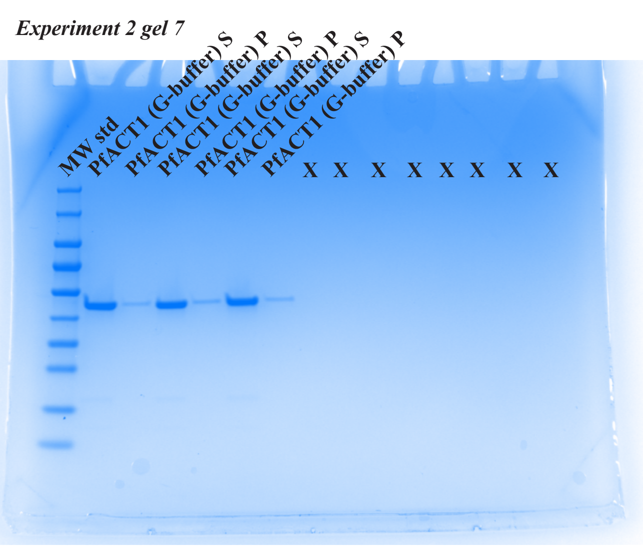

Experiment 3 gel 1

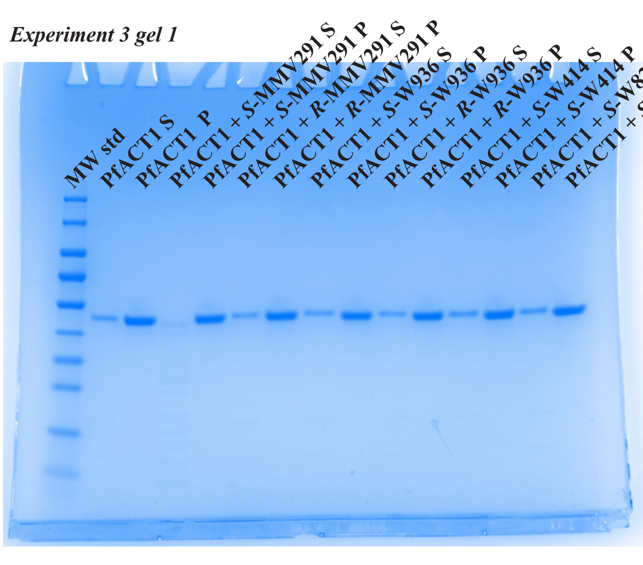

Experiment 3 gel 2

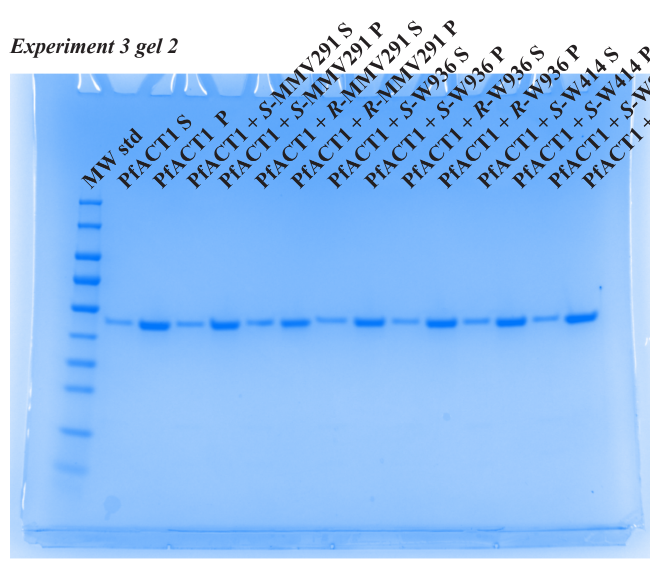

Experiment 3 gel 3

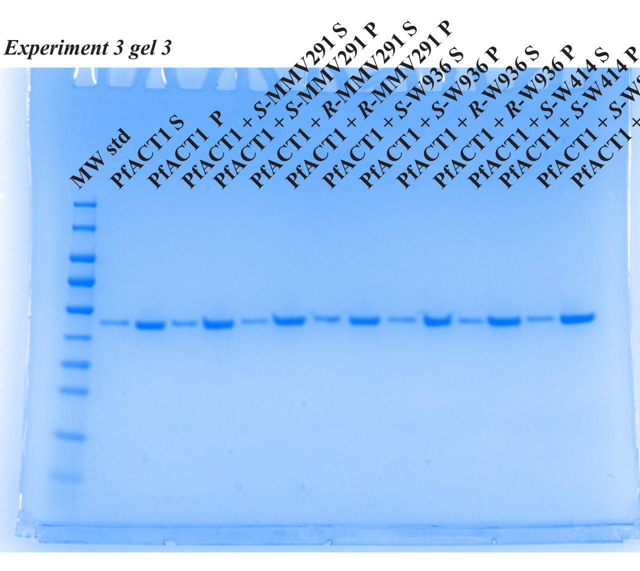

Experiment 3 gel 4

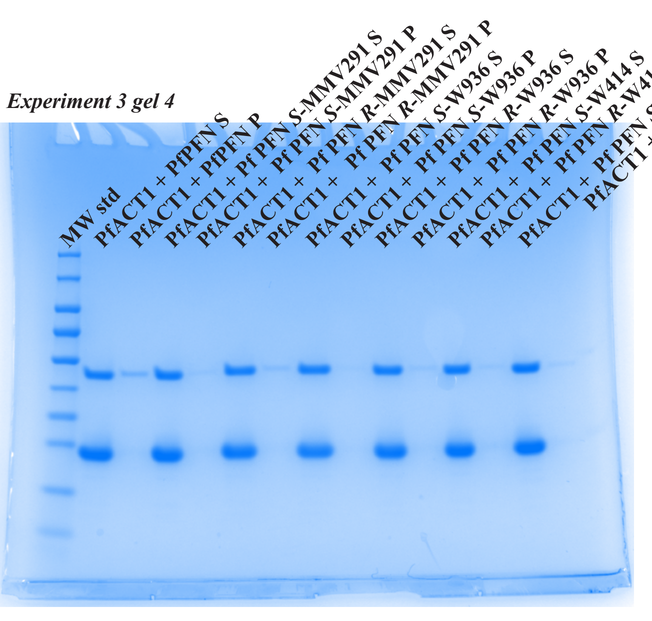

Experiment 3 gel 5

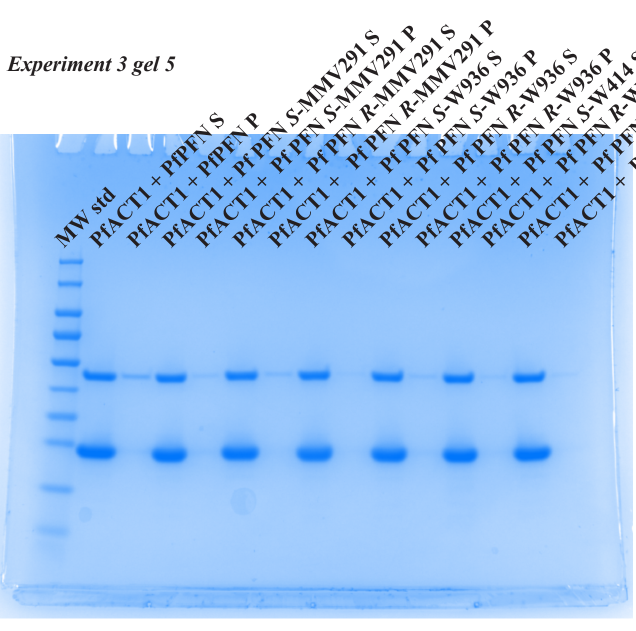

Experiment 3 gel 6

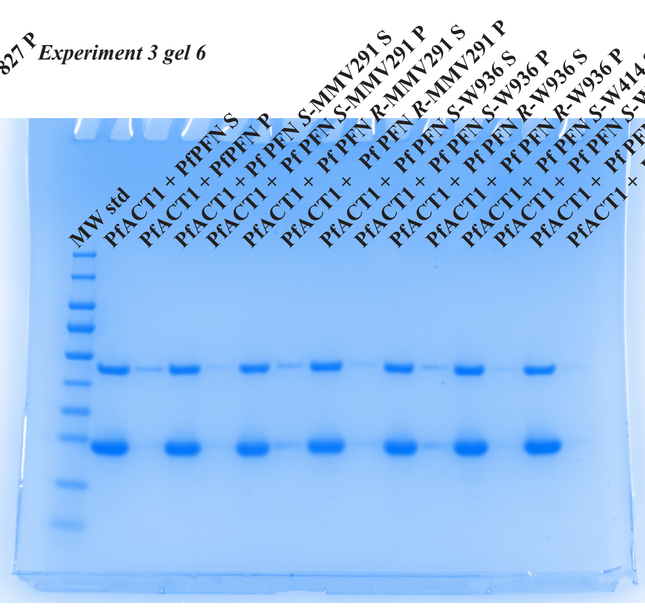

Experiment 3 gel 7

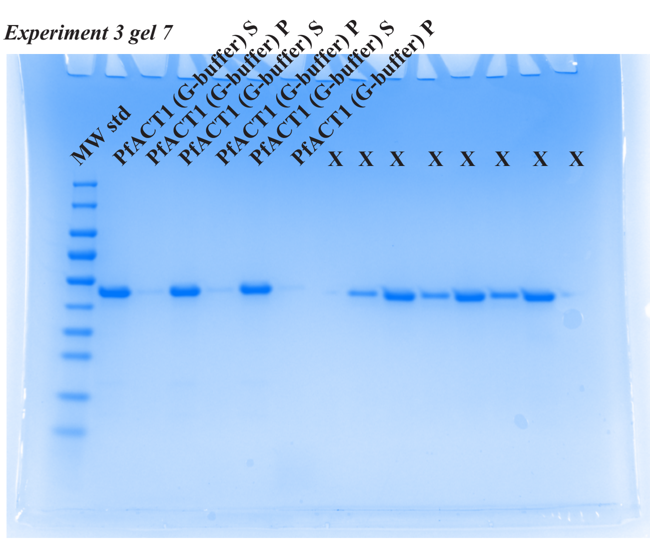

Experiment 4 gel 1

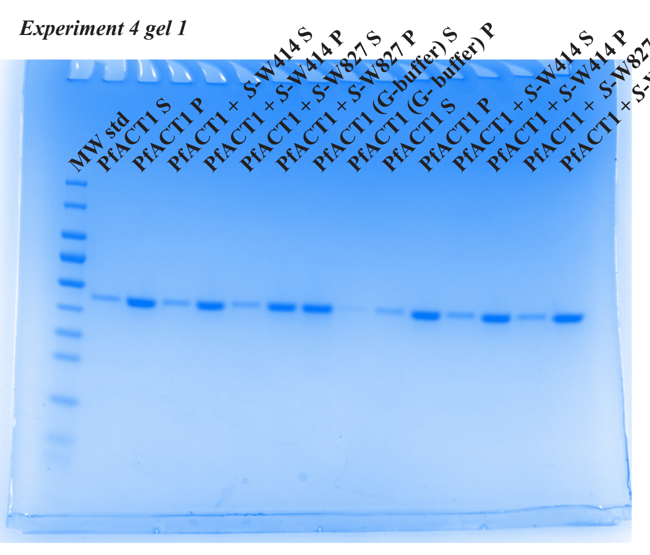

Experiment 4 gel 2

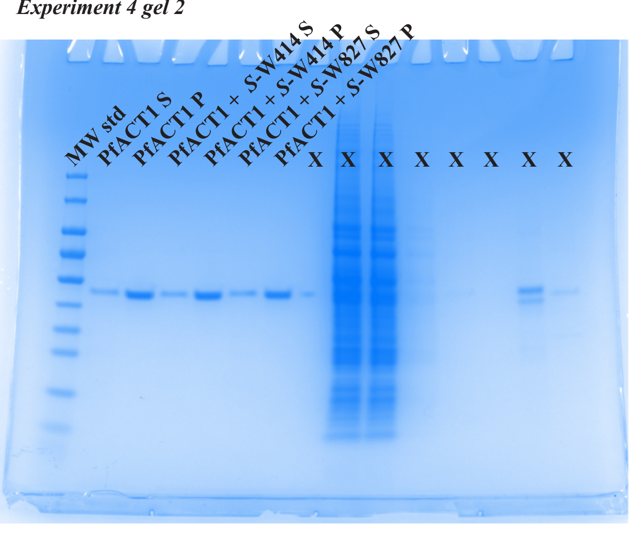

Experiment 5 gel 1

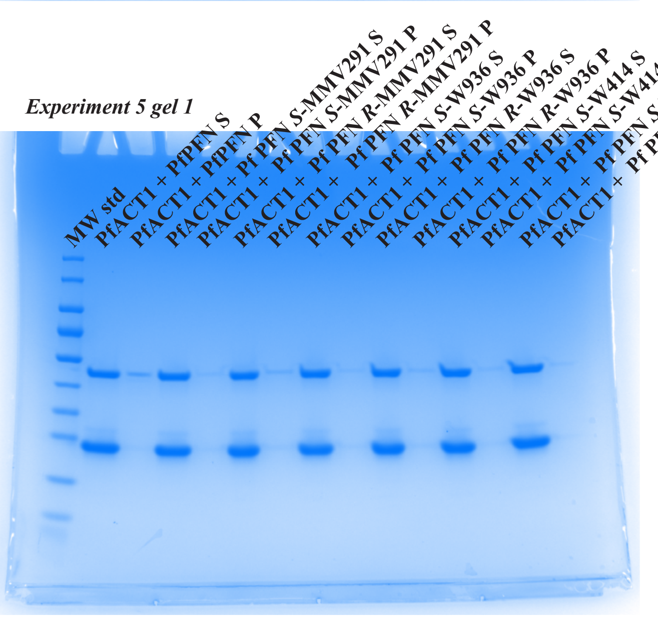

Experiment 5 gel 2

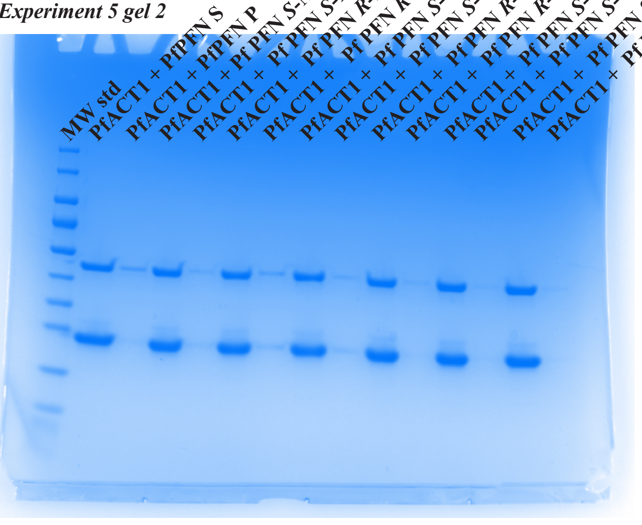

Experiment 5 gel 3

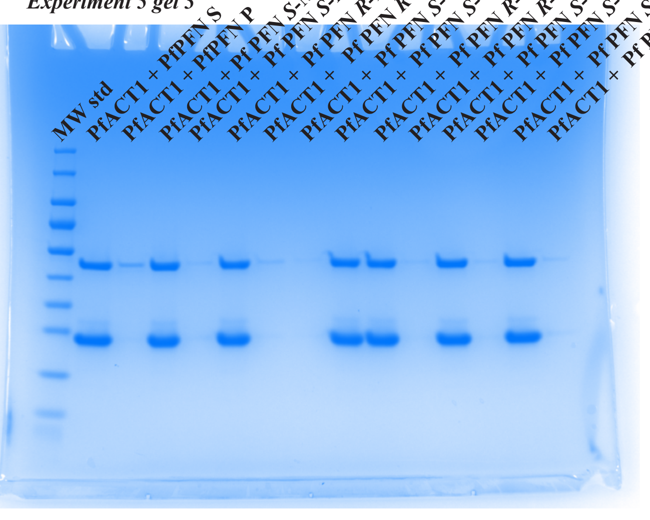

Experiment 5 gel 4

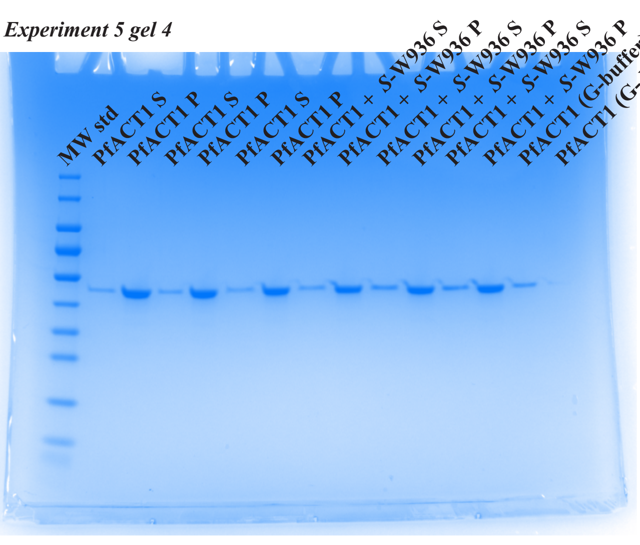

Experiment 6 gel 1

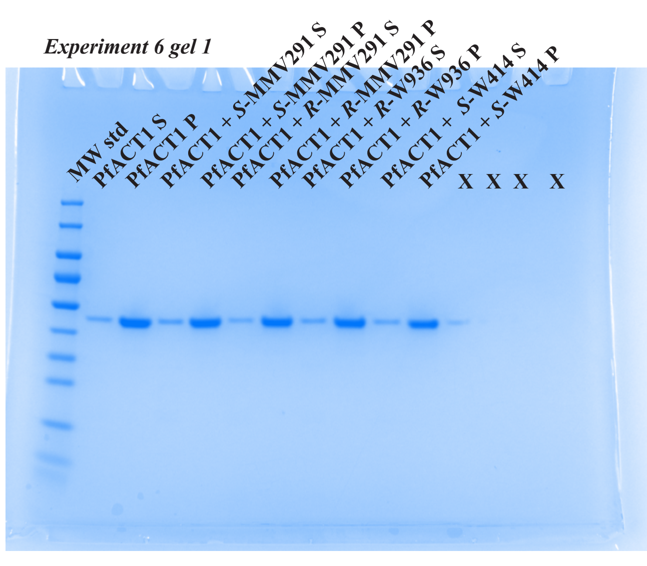

Experiment 6 gel 2

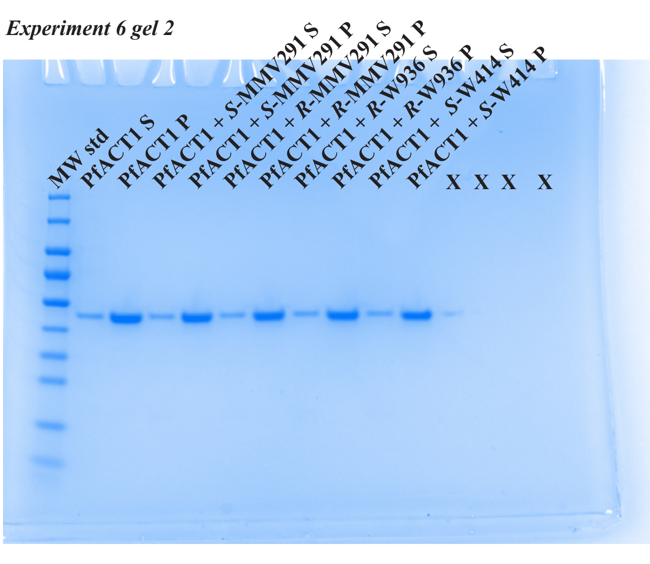

Experiment 6 gel 3

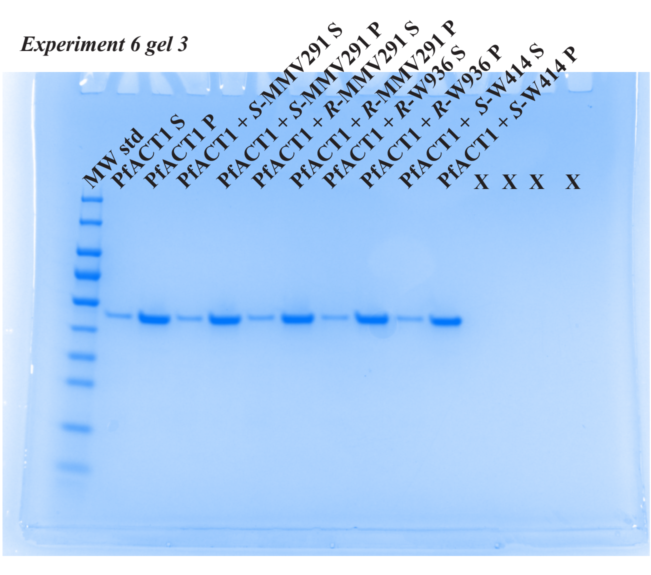

Experiment 6 gel 4

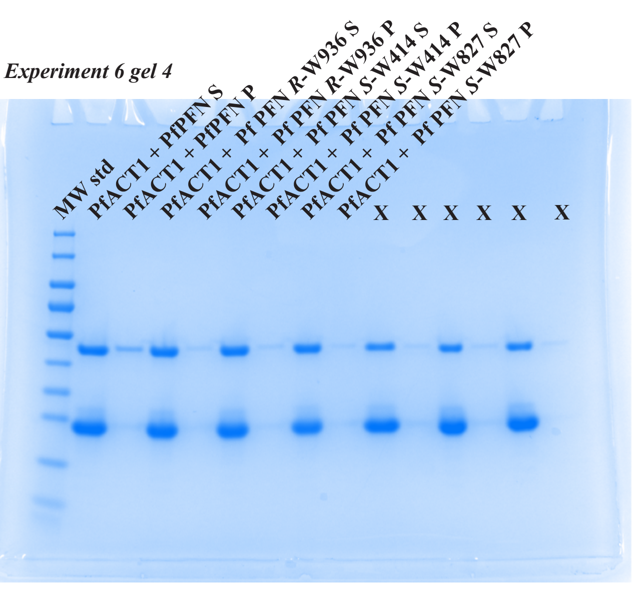

Experiment 6 gel 5

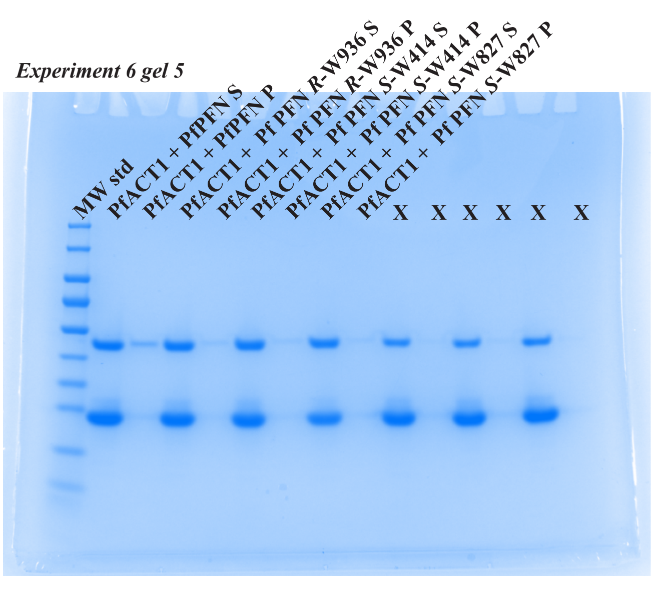

Experiment 6 gel 6

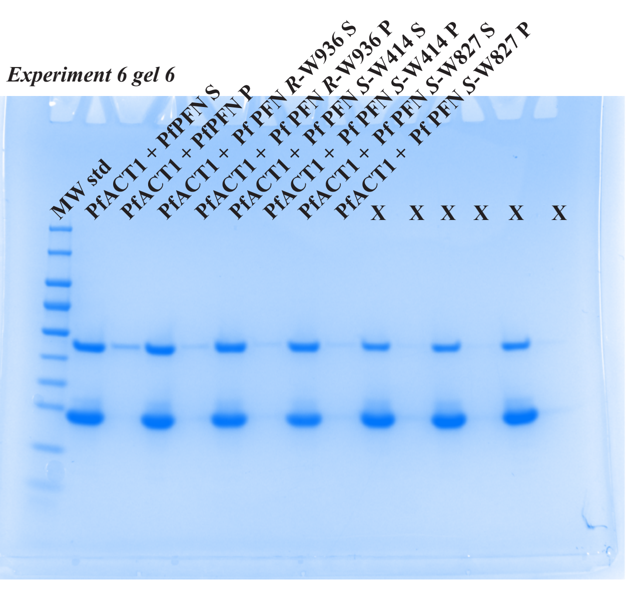

Experiment 6 gel 7

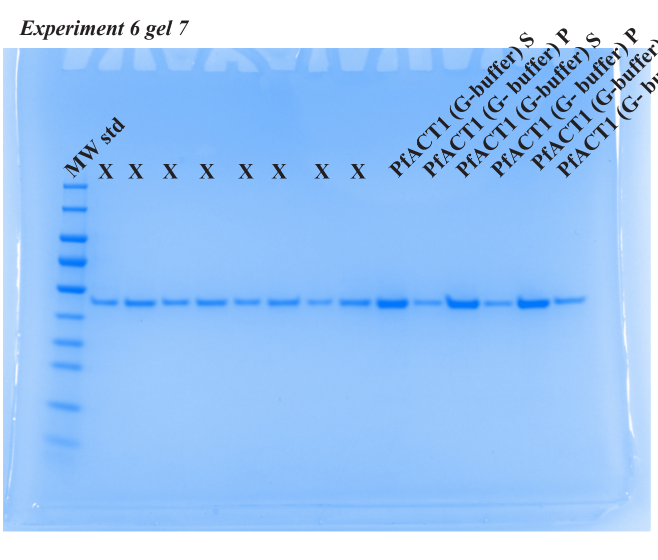

Supplement: S1 Raw Images — (PDF) [file pbio.3002066.s017.pdf]
