# Supplementary material for: Sulfonylpiperazine compounds prevent Plasmodium falciparum invasion of red blood cells through interference with actin-1/profilin dynamics
Source: PLoS Biol. 2023 Apr 13;21(4):e3002066. doi: 10.1371/journal.pbio.3002066 (PMC10128974; doi:10.1371/journal.pbio.3002066)
Supplement: Attachment — Submitted filename: Response to reviewers.docx [file pbio.3002066.s018.docx]

Please note all line numbers in this document refer to the revised manuscript file “Manuscript_revised with changes highlighted”

The World Malaria Report for 2021 has been published since submission of this manuscript so we have updated the number of deaths on line 49 as *“619 000 deaths in 2021”* and corresponding citation has been updated on line 940.

Reviewer #1: This is a convincing study that implicates actin-profilin dynamics in the mechanism of action of a potent compound series that blocks invasion of RBCs by Plasmodium falciparum.  The literature review is comprehensive and the data are largely supportive of the model proposed, with some limitations. Although it is clear that the compounds affect actin-profilin dynamics and disrupt F actin formation, it is less clear what the exact target of the compounds is.  This could be rectified by either performing the additional experiments described below or softening the conclusions about the nature of the "target".  In either case, the work is interesting and highly deserving of publication.

1)      There is somewhat of a disconnect between the selection of resistant mutants, the identification of mutations in actin and profilin, and the conclusion that these proteins are the "targets" of MMV291. My interpretation of "target" is that the compound binds directly to the molecule in question to alter its function, but I am not sure that is shown here. The inhibitor clearly alters the profilin-actin dynamics in vitro, and the mutants confer resistance in vivo, yet it is unclear if both molecules are "targets" in a classical sense. Data from the sedimentation data would support a direct interaction with the compounds and actin (since they increase turnover) but there is not a similar assay to show direct interaction with PFN.  Hence, it is possible the mutations in PFN simply alter interactions with actin, which is the direct target.  The authors favor a model where the mutations near the binding interface alter compound binding, but this is not shown directly.  As well,
the mutant PFN(K124) points away from the interface, so it must act allosterically. The authors should revise the text to be clear about what conclusions are really supported by the data and where they are speculating about mechanism. This issue affects the abstract, end of the introduction lines 222-223, and discussion.

We agree with these statements and need to be more precise with the use of the word “target” in the context of our study. As such, we have made the following changes to the manuscript as follows.

Line 27 (abstract): removed “*identify actin and profilin as* *putative targets*” and replaced with “*we generated resistance to MMV020291*”

Line 40 (abstract): changed “*targeting the actin-1/profilin interaction in P. falciparum*” to *“interfering with the actin-1/profilin interaction in* *P. falciparum”*

Line 133 (introduction): changed “*putative targets of MMV291 and PfPFN and PfACT1*” to “*the mechanism of resistance to MMV291 are through mutations in PfPFN and PfACT1*”

Line 135 (introduction): changed “*targeting the actin-profilin complex”* to “*linked to interference with the actin-profilin complex*”

Line 164 (Figure 1 legend): changed “*putative targets*” to “*protein candidates involved in the MoA*”

Line 201 (Table 1 legend): changed “*putative targets*” to “*protein candidates involved with the MoA*…”

Line 230 (results): changed “*the putative targets*” to “*involved in the MoA*”

Line 244 (Figure 2 legend): changed “*putative targets*” to “*involved in the MoA*”

Line 467 (discussion): changed “*putative targets”* to “*proteins involved in the MoA”*

Line 603 (discussion): sentence removed

Regarding the statement that PFN(K124) points away from the binding interface so it must act allosterically- these two proteins have not been solved in a bound state and therefore this is only a predictive model based on bound *O. cuniculus* actin and human profilin. PFN(K124), as presented in our model, is therefore only an approximate location and therefore could be involved in direct actin binding, or alternatively as the reviewer suggests, act allosterically.

Taking all of this together, we have added a discussion point on Line 512:

“*Whilst the predictive model of bound PfPFN and PfACT1 places two of the three resistance mutations in the binding interface between the proteins, the exact binding location and subsequent ‘target’ of MMV291 remains to be uncovered. Further studies into the compounds’ effects on the kinetics of actin polymerisation in parasites and crystallography studies solving the binding site of the MMV291 series in relation to the PfPFN-ACT1 interaction would be worthwhile attempting in order to gain a greater understanding of the druggable potential of these essential parasite proteins.”*

2)      The use of chromobodies to study polymerized actin is useful for monitoring the sensitivity to actin disrupting agents. However, this approach is somewhat artificial as it is almost certain that introduction of the chrombody alters actin dynamics and result in a more stable F actin pool in the parasite.  This limitation should be acknowledged.

We agree the chromobody system has its limitations and indeed Stortz and colleagues (2012) found that the introduction of an actin-chromobody construct caused the overall motility of *T.gondii* to increase but the expression had little effect on F-actin dynamics specifically (PMID: 28322189).

We only used the actin-chromobody experiment to examine F-actin puncta in merozoites expressing the chromobody with different drug treatments compared with a vehicle control. As we did not use the parasites for invasion or motility, we feel that it was not relevant to highlight potential limitations with this technology.

3)      The results of the in vitro sedimentation assays are open to alternative interpretations. Addition of the MMV analogs to sedimented actin decreases the amount of F actin from 80% to 68% in one case, which is statistically significant.  Addition of the compounds to a mixed fraction containing actin and PFN also further decreases the amount of F actin.  The authors interpret this result to show that the compounds stabilize the PFN-G-Actin interaction, thus resulting in less F actin. This result might be better supported by examining the ATP-ADP exchange rate of actin when bound to PFN in the absence vs presence of compounds.  Is there a direct effect on the stability of PFN-G-actin?  Alternatively, it seems equally plausible that the compounds increase the turnover of F actin, releasing G actin which is then bound by PFN. Sorting out these two models would be possible using more quantitative assays for actin turnover (pyrene actin polymerization followed by compound
addition and monitoring of stable actin) or for PFN nucleotide exchange, as suggested above.  In the absence of more definitive data, the authors should modify their conclusions to allow for both possibilities.  In particular, the Conclusion rather strongly states that there is no effect on actin polymerization directly, but the data on Figure 5 do not support this conclusion.

The increased turnover model is something we also have considered however we feel that our proposed stabilization model better fits the observed data for the following reasons:

1. As the reviewer pointed out, there was one compound (*S*-936) that statistically significantly reduced the % of actin in the pellet without PFN present. We believe that this is due to the greater error from a technical replicate in one of the biological repeats of the experiment and not a real result (*S*-936 Stdev= 6.9, remaining treatments Stdev <4.9). We acknowledged this significant reduction on:

Line 337: “*S-W936 was found to cause a small but significant reduction in the amount of PfACT1 in the pellet to 68%... These results indicate that the MMV291 analogues have either no or minimal impact on actin polymerisation in vitro*”

Line 494: “*the compounds had no activity against PfACT1 polymerisation in the absence of PfPFN, apart from S-W936 that caused a slight reduction*.”

2. The F-actin chromobody data better supports the stabilisation model rather than the turnover model because the compounds significantly reduced F-actin. If turnover for being affected, then there would still be significant F-actin present which was not observed.
3. The increased turnover model would mean that the resistant-inducing mutations in PFN would arise from some mechanism to “slow-down” this increased actin turnover to a homeostatic state (ie. perhaps stabilize PFN-G-ACT1 to deliver less G-ACT1 to the barbed end of the filament?). This would likely have a fitness cost to the parasite which we did not observe in these mutants. We feel that it is more likely that the resistant mutations prevent MMV291 interference in the protein-protein stabilisation of these two proteins.

However, as the reviewer points out, we do not have evidence to fully support our stabilisation model. We are planning further studies using techniques that the reviewer suggests to further explore this but in the absence of these in this manuscript, we agree that toning down our conclusion is warranted. As such, we have removed the “stabilisation” inference from the abstract and results section:

Line 35: now reads *“In vitro co-sedimentation experiments using recombinant P. falciparum proteins indicate that potent MMV020291 analogues disrupt the formation of filamentous actin in the presence of profilin”*

Line 352: removed the statement that the compounds stabilise the interaction and now reads: “*In summary, these results indicate that the compounds may act through a PfPFN-mediated mechanism to interfere with actin polymerisation in parasites*”

Line 367 (Figure 5 legend): removed “*indicating a stabilisation of the PfACT1-PfPFN complex*” and changed the title to read “*MMV291 analogues interfere with actin polymerisation in the presence of profilin in vitro*”

In the discussion, we have removed some references to the stabilisation as follows:

Line 502: removed “*of this stabilisation*”

Line 580: removed “*that stabilising the PfACT1-PfPFN interaction*” and replaced with “*this particular interference in the PfACT1-PfPFN interaction*”

In other instances, where appropriate to speculate about the MoA, we have emphasized that the possible stabilisation is only a theory:

Line 502: altered “*suggests that this compounds series stabilises the interaction…”* to “*suggests that this compound series could stabilise the interaction…*”

Line 509: addition of “*MMV291 may increase the PfPFN sequestering effect…”*

Line 512: addition of ““*Whilst the predictive model of bound PfPFN and PfACT1 places two of the three resistance mutations in the binding interface between the proteins, the exact binding location and subsequent ‘target’ of MMV291 remains to be uncovered. Further studies into the compounds’ effects on the kinetics of actin polymerisation in parasites and crystallography studies solving the binding site of the MMV291 series in relation to the PfPFN-ACT1 interaction would be worthwhile attempting in order to confirm this stabilisation model and gain a greater understanding of the druggable potential of these essential parasite proteins.”*

Line 529 (Figure 7 legend): B) now reads “*A potential* *mechanism for MMV291’s inhibitory activity could be through the stabilisation of the G-actin/profilin dimer therefore inhibiting the formation of F-actin and preventing the generation of force required for invasion*”

Line 577: changed “*that stabilisation of the PFN-ACT1…”* to “our proposed MMV291 MoA of stabilisation of the PFN-ACT1…”

Minor comments:

Line 95 reference not formatted

Corrected

Line 114 MoF define
 MoA is the abbreviation used on line 114 (now line 121) which is defined on line 55.

Reviewer #2: With 241 million cases and around 627 000 deaths, 77% of them concerning children aged under 5 years, malaria remains a devastating parasitic disease. Treatment failure due to emergence of artemisinin partial resistance increases the urgency for the development of novel parasiticidal drugs and more specifically drugs directed against new targets and with new mode of actions (MoA).
In this article Dans and colleagues characterize a series of new compounds targeting for the first time the actin-1/profilin complex and inhibiting the invasion of the red blood cell by the merozoite. This target was identified through the whole genome sequencing of chemically induced MMV291-resistant parasites and confirmed via reverse genetics. The MoA of the inhibitors was then dissected using biochemical approaches.
Overall, the manuscript is well-written, the experiments are clearly explained and well conducted with all the appropriate controls. The study is of high quality and of interest for a general audience since it identifies a new MoA. MMV291-based compounds stabilizes the interaction between Pfactin-1 and Pfprofilin, leading to a decrease of actin polymerization. In Apicomplexan parasites, actin filaments are made at the time of movement and are critical for invasion. Surprisingly the compound series is not acting on Toxoplasma gondii or other stages of Plasmodium falciparum.


I just have some issues concerning the delayed death phenotype experiments which I may not have understood correctly (Fig.6B, iii). How do the authors discriminate between an invasion phenotype and delayed death phenotype? In both cases, the biomass measured by nanoluciferase protein content would drop. Is it just because the drug was removed before merozoite invasion? Did the authors check the apicoplast morphology by immunofluorescence in the 2nd and 3rd cycle after a 40 h treatment stopped prior to merozoite invasion for 1 or 2 doses of MMV291?
It is somehow surprising not to have a stronger drop in the biomass if we compared to WT parasites of Fig. S1. The two experiments seem to be done with the same range of MMV291 concentrations but with a different assay (LDH vs nanoluciferase). Is it only due to different incubation time in presence of the compound?

The reviewer is correct in ascertaining that the drug was removed before merozoite invasion as referred to on line 437: “*After 40 hours and prior to merozoite invasion, the compounds were washed out and parasites allowed to grow for a further two cycles…”* as this allowed quantification of the compound’s effect solely on delayed death. This is a crucial aspect of the methodology of this assay and therefore to aid in the understanding of the experimental set-up, we have now included a diagram as (Figure 6B) (see below) prior to presenting the data from the assay (referred to on line 439 in-text).

In the nanoluciferase delayed death experiment, we did not check the apicoplast morphology after the 2^nd^ and 3^rd^ cycle with MMV291 as we had already shown a defect in apicoplast segregation after the 1^st^ cycle in Figure 6A.

The differences between the LDH and nanoluciferase growth assays are, as the reviewer pointed out, due to the differences in incubation times with the compound based on the different stages the parasites are exposed to. In the LDH assay, ring-stage parasites are incubated with the compounds for 72 hrs until trophozoite stage the following cycle, containing a cycle of merozoite egress and invasion and therefore capturing the invasion phenotype of the compounds. In contrast, the nanoluciferase experiment involved treating ring-stage parasites up until schizonts for 40 h before washing the compound out to evaluate the delayed death effect on intracellular growth. Therefore, as the nanoluciferase parasites never go through the invasion step in the presence of the compounds, the overall in effect in parasite reduction is much less than for the 72 hour LDH assay where they do go through the invasion step.

Reviewer #3: In this manuscript by Das and colleagues, the authors identified actin and profilin as putative targets of MMV291.
To identify these targets the authors generated MMV291 resistant parasites and identified 3 clonal lines with a mutation in actin (1 clone) or Profilin (2 clones). Replacing the endogenous genes with mutant variants, conserved resistance to MMV291.
The authors then undertake a series of experiments to investigate how MMV291 and analogs alter F-actin/profilin interaction and function. To summarize briefly the results - In vitro experiments with recombinantly purified Act1 and PFN demonstrate that PFN decreases the amount of F-actin in the same. The amount of F-actin is further reduced upon MMV291 treatment. In vivo, MMV291 alters actin organization in merozoites and apicoplast inheritance
These results are significant as they demonstrate that disruption of actin-pfn interaction is a potentially viable target for development of anti-malarial drugs. While this is an exciting prospect this claim is dependent on a MMV291 activity being specific for Plasmodium actin and pfn while not exhibiting inhibitor activity against human actin and profilin.  This was not established by the authors.  My second major concern is that authors repeatedly state that MMV291 increases actin sequestration by proflin but this is not directly demonstrated.

Major concerns:
1)      The authors claim on a number of occasions (Abstract line 36; results line 346; discussion line 482) that the changes in actin organization and function after MMV291 treatment is due to increased actin-PFN binding. The authors should show this directly using their previously established native gel assay (Moreau et al 2017). Use of more sophisticated actin polymerization assays such as pyrene-polymerization assays previously used by the authors or TIRF microscopy assay would yield greater insight into how MMV291 is altering actin nucleation and elongation.

We thank the reviewer for their suggestions and agree that using some of these techniques would more definitively show our predicted stabilisation model is the MoA for this compound series. We plan to complete these experiments in the next stage of our investigation into these compounds, along with crystallography trials. In the absence of these results for this manuscript, we agree that claiming the compounds MoA is through stabilisation of PFN-ACT1 complex is not warranted. As such, we have removed this inference from the abstract and results section from the sedimentation experiment (please note that the line numbers reflect the revised manuscript):

Line 35: now reads *“In vitro co-sedimentation experiments using recombinant P. falciparum proteins indicate that potent MMV020291 analogues disrupt the formation of filamentous actin in the presence of profilin”*

Line 352: removed the statement that the compounds stabilise the interaction and now reads: “*In summary, these results indicate that the compounds may act through a PfPFN-mediated mechanism to interfere with actin polymerisation in parasites*”

Line 367 (Figure 5 legend): removed “*indicating a stabilisation of the PfACT1-PfPFN complex*” and changed the title to read “MMV291 analogues interfere with actin polymerisation in the presence of profilin *in vitro*”

In the discussion, we have removed some references to the stabilisation as follows:

Line 505: removed “*of this stabilisation*”

Line 582: removed “*that stabilising the PfACT1-PfPFN interaction*” and replaced with “*this particular interference in the PfACT1-PfPFN interaction*”

In other instances, where appropriate to speculate about the MoA, we have emphasized that the possible stabilisation is only a theory:

Line 503: altered “*suggests that this compounds series stabilises the interaction…”* to “*suggests that this compound series could stabilise the interaction…*”

Line 509 addition of “*MMV291 may increase the PfPFN sequestering effect…”*

Line 512: addition of “*Whilst the predictive model of bound PfPFN and PfACT1 places two of the three resistance mutations in the binding interface between the proteins, the exact binding location and subsequent ‘target’ of MMV291 remains to be uncovered. Further studies into the compounds’ effects on the kinetics of actin polymerisation in parasites and crystallography studies solving the binding site of the MMV291 series in relation to the PfPFN-ACT1 interaction would be worthwhile attempting in order to confirm this stabilisation model and gain a greater understanding of the druggable potential of these essential parasite proteins.”*

Line 529 (Figure 7 legend): B) now reads “*A potential* *mechanism for MMV291’s inhibitory activity could be through the stabilisation of the G-actin/profilin dimer therefore inhibiting the formation of F-actin and preventing the generation of force required for invasion*”

2)      MMV291 activity was not shown to be specific for plasmodium actin and profilin. In vitro polymerization assays with purified mammalian actin and profilin should be used to determine the specificity of the compounds.

We agree with this statement and believe that this is an important question that needs to be addressed for the future development of compound series and are planning on conducting in vitro polymerisation assays with mammalian actin and profilin in the next stage of the study. However, to address this question experimentally in a timelier fashion, we have conducted lattice light shield imaging on HeLa cells that have been treated with MMV291 or other actin inhibitors and filaments have been visualized with an F-actin stain. This showed that MMV291 (even up to 20 µM) has no effect on actin filaments compared with CytD and Lactrunculin B treatments. The following figure is now included as Supplementary Figure 13 and is referred to in-text on lines 447-458 in the results section and line 547 and 555 in the discussion section.

A/Prof. Kelly Rogers and her team who are experts in lattice light shield imaging (Scally et al 2022 Nat. Microbio (PMID: 36396942), Geoghegan et al 2021 Nat. Comms (PMID: 34131147), Pasternak et al 2022 Commun. Biol (PMID: 35393572), Gan et al 2022 Nature (PMID: 35293391)) have now been included in the authorship for their contribution to the manuscript with this data.

3)      Chromobody images are low resolution and out of focus. Authors should provide images of higher resolution

We are unsure why the image quality is not satisfactory, it was possible that during generation of the PDF for submission, the file was compressed and lost resolution. We have also re-examined the original images and have chosen to show alternative fields of merozoites in the figure below for MMV291 (2 µM) and *R*-W937 (1 µM) treatments that appear a bit sharper.

4)      Authors need to include detail microscopy methods for chromobody imaging - Minimum information needed includes microscope make and model, camera make and model, lens information (magnification and NA), and filter sets.

This information has now been added to the methods section on line 826:

“*Parasites were imaged using the Leica DMi8 widefield microscope attached to a Leica DFC9000 GTC camera, using a 100x objective.”*

5)      Toxoplasma invasion assay is novel and not standard in the field. Authors should provide key controls that extracellular parasites are effectively washed off from the monolayers and are not significantly contributing to the assay. Secondly, the parasites invaded host cells in the presence of compounds. Then compounds were washed out and parasites grown for 24 hours post invasion. Authors should state if the inhibitory effect of these compounds are reversible and include appropriate references. If the effects are irreversible, authors should provide controls to demonstrate whether the compounds affect intracellular replication.

This assay is standard in the field, for example; Carey et al PNAS 2004 (PMID 15123807), Lourido et al 2010 Nature (PMID: 20485436), Lourido et al 2012 EMBO (PMID: 2319386). Our detailed protocol is based on that described in Lourido et al 2012 EMBO but incorporates nanoluciferase as a method of detection. In this assay, the washing off of extracellular parasites can be seen to be effective as otherwise you would not see the dose-response inhibition trend we visualize in this experiment with our compounds.

To address the second point, the genetic knockout of actin in Toxoplasma does have a delayed death effect in parasite replication (due to preventing apicoplast segregation), but not in the time frame that we used (24hrs) (Egarter et al, PLoS One, PMID: 24632839). It can therefore be concluded that the compounds effect on actin at high concentrations (even if they were not reversible) could not be due to effects during replication.

Minor Concerns:
Line 77: The definition of treadmilling is incorrect.

We have removed the definition stated on line 77 (now line 83) and instead have defined actin treadmilling on line 87 to read as follows:

*“The continuous growth at the barbed end and shortening at the pointed end of the filament is referred to as actin treadmilling (25)…”*

Line78: ATP hydrolysis occurs once G-actin has been incorporated into the filament. As written G-actin-ATP to G-actin-ADP is misleading. This sentence should be re-written.

We agree with this statement and have amended the section from line 80-87 as follows:

*“F-actin is formed through the incorporation of subunits of globular actin (G-actin)-ATP at the barbed end. To stabilise the growing filament, hydrolysis of G-actin-ATP occurs to generate G-actin-ADP and inorganic phosphate (P_i_) (24-26). For filament disassembly and G-actin turnover, the release of P_i_ at the pointed end destabilises the F-actin, resulting in the disassociation of G-actin-ADP (27, 28).”*

Line 95: Moreau et al 2017 reference is not formatted

Corrected

Line 114: Write out acronym MoA at first use

MoA is defined on line 55
